# Auchenorrhyncha (Hemiptera) from Crete deposited at the Natural History Museum of Crete

**DOI:** 10.3897/BDJ.14.e177170

**Published:** 2026-01-30

**Authors:** Radost Angelova, Ilia Gjonov, Apostolos Trichas

**Affiliations:** 1 Sofia University "St. Kliment Ohridski", Sofia, Bulgaria Sofia University "St. Kliment Ohridski" Sofia Bulgaria; 2 Sofia University, Faculty of Biology, Sofia, Bulgaria Sofia University, Faculty of Biology Sofia Bulgaria; 3 Natural History Museum of Crete, Heraklion, Greece Natural History Museum of Crete Heraklion Greece

**Keywords:** Cicadomorpha, Fulgoromorpha, Greece, museum collection

## Abstract

**Background:**

Crete, the largest island in Greece, is characterised by its diverse landscapes, ranging from coastal ecosystems to high-altitude mountain ranges. Its complex geological history, Mediterranean climate and varied vegetation types have contributed to a rich and unique biodiversity, including numerous endemic species. These environmental factors create favourable conditions for the diversification of insect fauna, including members of the suborder Auchenorrhyncha.

Auchenorrhyncha are sap-feeding insects within the order Hemiptera. They are divided into two major infraorders: Cicadomorpha and Fulgoromorpha. According to published records, the Auchenorrhyncha fauna of Crete includes two families from the superfamily Cercopoidea — Cercopidae with one species and Aphrophoridae with four species — along with the superfamilies Cicadoidea (1 family, 3 species) and Membracoidea (1 family, 75 species), all of which belong to Cicadomorpha (83 in total). The infraorder Fulgoromorpha, distinct from the aforementioned group, has 57 species across seven families. In total, 140 species of Auchenorrhyncha have been recorded on the Island.

**New information:**

An analysis of 2,849 specimens from over 400 localities in Crete, housed at the Natural History Museum of Crete (NHMC), revealed 93 Auchenorrhyncha species across 10 families, representing approximatley 64% of the Island’s known fauna. Despite limitations in sampling methods, 37 species were newly recorded for Crete, 10 of which are also new to Greece and 13 genera were reported from Crete for the first time. The Island’s known Auchenorrhyncha fauna now totals 177 species, underscoring the importance of museum collections and continued entomological research. Additionally, an updated checklist of the Auchenorrhyncha in Crete is provided as supplementary material.

## Introduction

Crete is the largest Greek island and the one situated closest to North Africa. It occupies a unique biogeographic position at the crossroads of Europe, Asia and Africa, contributing to a distinctive blend of flora and fauna from all three continents. The Island's rich and diverse flora and fauna are the result of its long-term geographic isolation, complex topography and mild Mediterranean climate. These factors have created a mosaic of microclimates that support a high number of rare and endemic species (Lazarina et al. 2019). Over 2,000 vascular plant species have been documented on Crete, approximately 14% of which are endemic (Lazarina et al. 2019, Menteli et al. 2019). Crete is also a major migratory corridor for birds and a key area for biodiversity conservation in the Mediterranean Region (Vogiatzakis et al. 2020).

The natural vegetation of the Island primarily consists of sclerophyllous evergreen forests — mainly *maquis* and *phrygana* — covering only about 2% of the Island’s surface, while the mountainous areas are dominated by dwarf shrublands (Zimowski et al. 2013). Crete is divided into four regional units (nomoi), arranged from west to east: Chania, Rethymno, Heraklion and Lasithi. Heraklion is the largest city and functions as the Island's capital. To the north lie the islands of Dia and the Dionysades group, while to the south are Koufonisi, Gaidouronisi, Paximadia, Gavdopoula and Gavdos - the southernmost point of Greece (Vogiatzakis and Rackham 2008).

The Island is traversed by several mountain ranges from west to east: the Lefka Ori (2,454 m), Kedros (1,777 m), Ida or Psiloritis (2,456 m), Kofinas (1,231 m), Dikti (2,085 m), Thripti (1,476 m) and Ornos (819 m). These Cretan mountains, part of the Dinaric system, are marked by steep slopes, deep gorges and abundant karst formations. Combined with caves and isolated habitats, they further enhance the island’s ecological diversity. The entire Island lies within a highly seismic zone. Major agricultural crops include grapevines, olive, orange and fig trees (Grove and Rackham 1993, Legakis et al. 1993). The long-term isolation, absence of large predators and limited colonisation by mainland species have led to the evolution of highly adapted, often endemic fauna. This is especially true for invertebrates, such as insects, where phytophagous groups like Auchenorrhyncha exhibit notable diversity and specialisation towards local flora.

Auchenorrhyncha, a suborder of Hemiptera and part of the hemimetabolous insects (Hemimetabola), includes four monophyletic suborders: Sternorrhyncha, Auchenorrhyncha, Heteroptera and Coleorrhyncha. Their phylogenetic relationships remain under debate, although Auchenorrhyncha is believed to have originated in the Early Permian (Holzinger et al. 2003). It comprises two main infraorders: Cicadomorpha and Fulgoromorpha. The former includes the superfamilies Cercopoidea (spittlebugs), Cicadoidea (periodical cicadas) and Membracoidea (with the families Membracidae and Cicadellidae), while Fulgoromorpha includes superfamilies Fulgoroidea with 17 families, Delphacoidea with three families and Meenoploidea with two extant families. Globally, over 47,000 species have been described (Dimitriev et al. 2022), with numbers growing annually. Most species feed on plant sap — either from the phloem, xylem or parenchyma — though some nymphs of Derbidae and Achilidae feed on fungal hyphae (Nickel 2003, Dietrich 2009, Bartlett and Wilson 2023). They are predominantly mono- or oligophagous, with a few exceptions (Denno and Perfect 1994, Nickel 2003).

To date, the Auchenorrhyncha fauna of Crete include representatives of the superfamilies Cercopoidea, Cicadoidea and Membracoidea. Cercopoidea is represented by one species from the family Cercopidae and three from the family Aphrophoridae. The superfamily Cicadoidea comprises two families with a total of seven species. However, four of these *Aestuansella aestuans* (Fabricius, 1794) (Lucas 1854), *Cicada orni* Linnaeus, 1758 (Haupt 1928), *Dimissalna dimissa* (Hagen, 1856) (Haupt 1928, Nast 1972) and *Oligoglena tibialis* (Panzer, 1798) (Haupt 1928, Nast 1972) were not confirmed in recent studies and their earlier records are considered doubtful, as previously noted by Quartau and Simões (2005) and Trilar et al. (2020). Superfamily Membracoidea is represented solely by the family Cicadellidae with 75 recorded species. The infraorder Fulgoromorpha comprises 57 species across seven families, making a total of 140 documented Auchenorrhyncha species on the Island. The first recorded species from Crete was *Issus palipes* Lucas, 1853 (Issidae), later reclassified as a likely nymph of the genus *Tettigometra* (Tettigometridae) by Gnezdilov (2017), based on Lucas’s original illustration and description.

In this study, a total of 2849 specimens of Auchenorrhyncha, collected from more than 400 different localities on the island of Crete and deposited at the Natural History Museum of Crete, were examined. A portion of the material was derived from soil traps during earlier surveys conducted between 1988 and 2018 and was previously identified to the order level. Additional specimens were collected by the first author using sweep netting and aspirator during fieldwork from May to September 2023.

The present study identified 93 species belonging to 11 families of the suborder Auchenorrhyncha and deposited in the collection of the Natural History Museum of Crete. These account for approximately 64% of the known Auchenorrhyncha species from Crete. Amongst the identified taxa, 37 species are newly recorded for Crete, 10 of which are also new to Greece and 13 genera are reported for Crete for the first time, nine of the species present in the collection are considered as endemic for the island and another four are endemic for Greece. A dataset containing the occurrence records for all specimens has been published on the GBIF (Angelova et al. 2025a).

## Materials and methods

### Survey Period

The present study includes 2849 specimens from over 400 different localities on the Island of Crete, deposited in the Natural History Museum of Crete. Most of the material was obtained from pitfall traps collected during previous research by the Natural History Museum of Crete, gathered between 1988 and 2018 and another part was collected by the first author between May and September 2023 using sweep netting with an entomological net and aspirator.

### Survey Areas

The material comes from diverse habitats and altitudes on the Island of Crete and adjacent smaller islands, such as Dia, Gavdos, Gavdopoula and Elafonisi. Complete information on the collection sites is provided in a GBIF dataset (Angelova et al. (2025), https://doi.org/10.15468/xh2zmd).

### Field Methods

**Pitfall Trap Method**

The zoological material from the alcohol collection of the Natural History Museum of Crete was collected using pitfall traps and sorted to the order level, stоred in ethanol (75-96%). Standard-sized cups (9.5 cm in diameter and 12 cm in height) were used, placed following a standardised setup. Ethylene glycol and undiluted propylene glycol were used as killing/preserving agents. Occasionally, small amounts of attractants like vinegar were used, along with liquid soap to reduce surface tension. Large stones were placed over the traps to prevent damage from grazing animals (flocks of sheep and goats on the Island). A more detailed description of the pitfall trap methods applied by NHMC between 1988 and 2018 is provided in Salata et al. (2020) and references therein.

**Sweep Netting with Entomological Net**

Material collected using sweep netting and an aspirator. The net consisted of a steel ring with a cotton bag, based on some Czech models, with a ring diameter of 35 cm. The handle was improved to be detachable into three parts for easier transportation. The aspirator was made using a cork stopper, a stainless steel inlet tube (220 mm long, 5 mm outer diameter) and a shorter outlet tube with an attached hose equal in length to an arm. Both tubes pierced the cork, which fitted tightly into the neck of a 50 ml centrifuge tube (30 mm diameter, 120 mm long). Inside the tube, a cotton plug was placed to provide a larger contact surface, preventing smaller insects from becoming overly moist and structurally damaged. A thin, long strip of folded filter paper was also added to absorb excess moisture and separate the interior space of the tube. Ethyl acetate was applied through the inlet tube, followed by inserting a cotton plug soaked with a few drops of the substance. Following a period of approximately 24 hours exposure to ethyl acetate vapours, the material was subjected to desiccation and stored until it was mounted on cotton layers.

### Laboratory Methods

Dry specimens, collected by sweepnet and aspirator, were rehydrated before mounting in a small container with a few drops of water. Alcohol-preserved material was prepared after drying on filter paper.

**Mounting**

Each specimen was glued with entomological adhesive to a properly-sized mounting card, then mounted on an entomological pin in a position allowing observation of key taxonomic and sexual features. Male genital segments were dissected and placed in a drop of distilled water (or 10% sodium hydroxide solution) in a small flat-bottomed test tube for a few minutes to macerate. This allowed for the safe opening of the genital capsule and easier removal of fat and muscle tissue and, after that, all of the structures were glued on the same mounting card as the specimen. Preparation was done under a Leica M60 stereomicroscope at suitable magnification.

### Collection Organisation

Specimens from the Natural History Museum of Crete were recorded in the Museum's internal database. Museum-provided labels were attached to the pin of each specimen. Each had two labels: the upper indicating sex and the lower containing collection info. Specimens were arranged in taxonomic order in wooden boxes with glass lids. The material is stored at the Natural History Museum of Crete (NHMC), curated by Apostolos Trichas PhD. A dataset has been generated containing metadata for all specimens in the collection. The dataset has been published on the GBIF platform and is accessible via https://doi.org/10.15468/xh2zmd (Angelova et al. 2025a).

### Spatial Distribution Analysis and Map Creation

The CSV file from the database was used as a layer in the open-source GIS software QGIS 3.22.4 Białowieża for spatial analysis. An OpenStreetMap layer was preloaded as the base map, with a shapefile overlay showing the borders of the four administrative regions of Crete – Chania, Rethymno, Heraklion and Lasithi.

### Results

**Figure 1.**
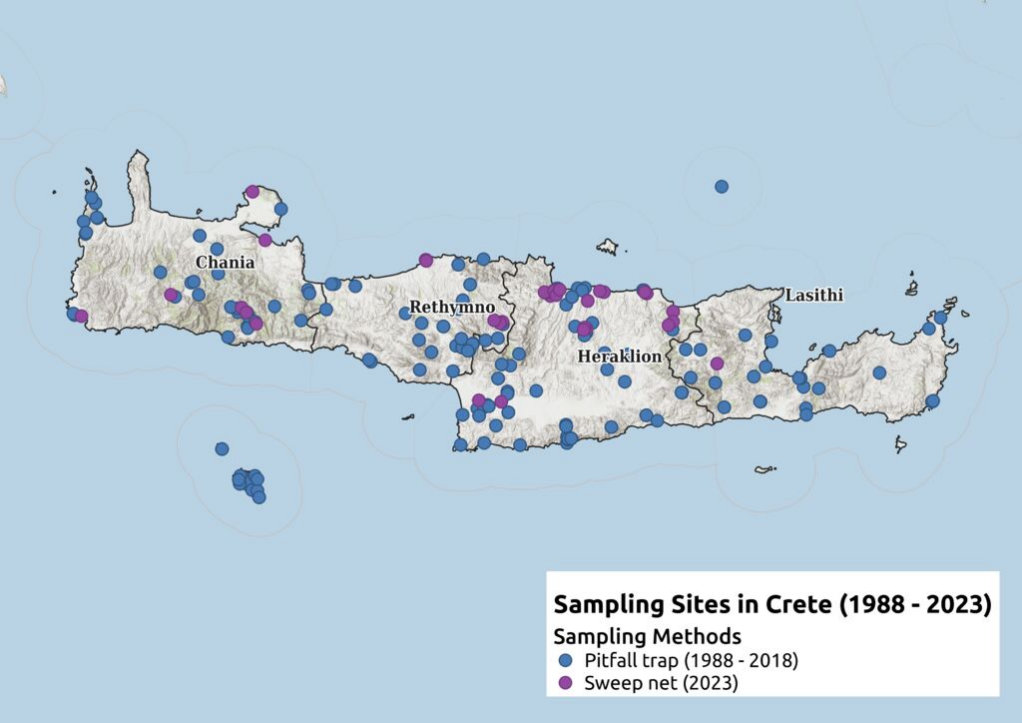
Sampling sites in Crete.

An analysis of specimens housed at the Natural History Museum of Crete (NHMC) involved the study of 2849 specimens from over 400 localities across the Island (Fig. 1). A total of 93 Auchenorrhyncha species were identified and prepared, representing 10 different families. These specimens represent 52.5% of the known species for the Island, demonstrating the value of the collection despite the challenges posed by the primary collection method (soil traps) and the limited duration of sampling with the most effective method (sweeping with an entomological net).

Amongst the identified species, 37 are newly recorded for Crete, 10 of which are also new to Greece. Additionally, 10 genera are reported for Crete for the first time and the total number of recorded Auchenorrhyncha species on Crete has increased to 177. These findings highlight the importance of museum collections in documenting insect diversity and underscore the need for continued entomological research to uncover the full extent of Auchenorrhyncha diversity on the Island.

The species included in the collection of the Natural History Museum of Crete are presented in taxonomic order. Synonyms under which each species has previously been reported for the Island of Crete are indicated. Hyperlinks with identifiers to two major databases - GBIF and 3I World Auchenorrhyncha Database (Dimitriev et al. 2022) - are provided. Localities are listed first according to literature records, where available, followed by newly-collected material. Additional notes are included regarding collection details, collection method, phenology, elevation and remarks on species distribution. Species newly recorded for Crete are marked with an asterisk (*), while species new to Greece are indicated with two asterisks (**). An updated checklist of the Auchenorrhyncha species of Crete is provided as a supplementary file (Suppl. material 1).

## Checklists

### Auchenorrhyncha

#### Fulgoromorpha

Evans, 1946

CD0A1194-FEB8-5C7D-9936-3F27DC7EB179

#### Fulgoroidea

Latreille, 1807

5A46408F-2B3E-5D72-A2A1-F73F73111059

#### Achilidae

Stål, 1866

2DE5E10C-C33F-5AC7-A2A7-D75C3AA32E49

#### Achilinae

Stål, 1866

42B1CB94-383E-5A6D-84F3-55A96A588B2B

#### Achilini

Stål, 1866

31EEB635-10A7-5CA9-A313-0F40FD49D5C7

#### Cixidiina

Emeljanov, 1993

40D9A274-6567-54CC-8F35-36AF9E1A18B1

#### Cixidia

Fieber, 1866

67FB8725-4354-5542-8359-E92D19788E4E

#### Epiptera

Metcalf, 1922

8E2565CF-8F92-539F-BCB2-675568C3B2D7

#### Cixidia (Epiptera) skaloula

Asche, 2015

1B0F3D56-6694-5A85-BBC5-C69581650A83

https://www.gbif.org/species/12178314

https://hoppers.speciesfile.org/otus/1032423

##### Distribution

**Literature data**: no specific location (Asche 2015). **New data**: Chania: Lake Agia.

##### Notes

Collected in pitfall trap from May to June at 44 m a.s.l. There are reports that the species occurs under the bark of decaying trees, where it probably feeds on the hyphae of saprotrophic fungi in the wood (Asche 2015).

#### Neomenocria

Fennah, 1950

139033D5-4C88-59A2-8C8C-DCDF8FF267C5

#### Neomenocria creticola

Asche, 2015

96AAC6C6-EDC7-5378-9521-E5BF952F1133

missing

https://hoppers.speciesfile.org/otus/1032426

##### Distribution

**Literature data**: Lassithi: Agios Ioannis (Dlabola 1977); no specific location (Dlabola 1989, Asche 2015). **New data**: Chania: on the road from Mochlos to Sfendili.

##### Notes

Considered endemic to Crete (Asche 2015). The species was collected by sweep netting in mid-June on the low branches of trees of the genus *Quercus* at 463 m a.s.l.

#### Issidae

Spinola, 1839

B7DB5A5F-AFFE-5D73-9760-92BFEDA9BF42

#### Hysteropterinae

Melichar, 1906

47987A61-6097-53C3-B23F-893266E3D653

#### Hysteropterini

Melichar, 1906

E8572884-07B7-5ED1-8146-5156C01F6DB9

#### Acrestia

Dlabola, 1980

FBA94B12-B6B7-5FD1-BCD3-158D5E111316

#### Acrestia

Dlabola, 1980

B6704D4A-BEE5-52FE-A2F3-73DE23D71DEB

#### Acrestia (Acrestia) suturalis

(Fieber, 1877)

839368D4-9E0A-5E02-8DA7-1F26535F4C5F

https://www.gbif.org/species/9370986

https://hoppers.speciesfile.org/otus/73233

Bubastia (Acrestia) suturalis (Fieber, 1877) in Gnezdilov et al. (2004).

##### Distribution

**Literature data**: no specific locations (Gnezdilov 2016). Chania: Therissos; Pithari; Lefka ori (Gnezdilov et al. 2004)**. New data**: Chania: Lefka Ori peak Troharis; Lake Kournas; Rethymno: Psiloritis, above Anogia on route E4. Heraklion: Koudoumas; Sterne; Giouchtas Mt.

##### Notes

Collected in pitfall traps left throughout the year up to 2347 m a.s.l. and by sweep netting in July and August on dry grasses up to 1111 m a.s.l.

#### Agalmatium

Emeljanov, 1971

B0C4D558-0224-5491-AD27-E96A1166B393

#### Agalmatium bilobum

(Fieber, 1877)

A3A880D5-2CE1-5E4A-9389-46EC1803A523

https://www.gbif.org/species/2051533

https://hoppers.speciesfile.org/otus/73244

##### Distribution

**Literature data**: surroundings Heraklion (Gnezdilov et al. 2004). **New data**: Chania: Gramvousa Peninsula; Gavdopula Island; Kouroupitos. Rethymno: Petres dunes; Mikronisi Island. Heraklion: Almyros River park; Gazi; Karteros; Lavris; on the road from Stalida to Mochlos; Rufas; Voroi; Platanos; Koudoumas; along the Almyros River; Kofinas; Siva; on the road from Mochlos to Sfendili; Aposelemis Dam; Sternes. Lasithi: Dikti Mountain; Xerocampos; Bramiana Dam; Mochlos; Pahia Amos.

##### Notes

Collected in pitfall traps for periods: from March to May and from May to August, collected by sweep netting from May to July. In the altitude profile – from sea level to 1105 m a.s.l. Occurs in dry habitats, polyphagous (Gnezdilov and Brien 2006).

#### Clybeccus

Gnezdilov, 2003

7CED9D9C-8AD3-56AF-AD4E-39D8B971AAA0

#### Clybeccus declivum

(Dlabola, 1986)

DF8B5243-F214-55D4-AA3A-D5118ACBF52D

https://www.gbif.org/species/7855000

https://hoppers.speciesfile.org/otus/73454

##### Distribution

**Literature data**: Rethymno: Agia Galini (Gnezdilov 2011) and no specific locations (Gnezdilov et al. 2014). **New data**: Heraklion: Aposelemis wetland. Lasithi: Xerocampos.

##### Notes

Collected in pitfall traps from May to August near the coast at 1-2 m a.s.l.

#### Latilica

Emeljanov, 1971

1E093D19-9DDF-512B-B10D-F46D0D04C2AD

#### Latilica oertzeni

(Matsumura, 1910)

A7E63104-D6CC-5839-AAB1-B6BFB8B26BA1

https://www.gbif.org/species/5982082

https://hoppers.speciesfile.org/otus/73310

##### Distribution

**Literature data**: no specific locations (Gnezdilov 2010, Gnezdilov et al. 2014). **New data**: Chania: Gavdos Isl.

##### Notes

Collected in pitfall traps from June to August on the northwest coast of Gavdos Island – 8 m a.s.l.

#### Latilica tunetana

(Matsumura, 1910)

EB52CA48-A669-5ACF-A9B6-B39AC30865F9

https://www.gbif.org/species/5982078

https://hoppers.speciesfile.org/otus/73848

##### Distribution

**Literature data**: Chania: Moni Gonias; Lassithi: Gra Ligiots (Gnezdilov 2010) no specific locations (Gnezdilov et al. 2014). **New data**: Chania: Gavdos Isl.; Lefka Ori between Anopoli and Pachnes Peak; Stavros, Zorbas Beach; Lake Kournas. Rethymno: Grazo; Heraklion: Martsalo; Rufas; Petrokefali; near the Almyros River; Voroi; Siva; Gazi. Lasithi: Paheia Amos; Xerokampos.

##### Notes

Collected in pitfall traps left throughout the year up to 800 m a.s.l. and by sweep netting in June and July up to 116 m a.s.l.

#### Mycterodus

Spinola, 1839

4B994A98-7DEC-5E7B-BE7F-C9A62BA35C11

#### Mycterodus

Spinola, 1839

6EC5F676-F64C-5386-8DA2-E8367424C4E4

#### Mycterodus (Mycterodus) lapaceki

Dlabola, 1984

A5AA1C32-4EA7-53E9-92BB-0ECD613792AC

https://www.gbif.org/species/4489714

https://hoppers.speciesfile.org/otus/73970

##### Distribution

**Literature data**: Chania: Hora Sfakion (Dlabola 1984) without specific locations (Gnezdilov et al. 2014). **New data**: Chania: Lefka Ori, Troharis peak; Heraklion: Koudoumas; Kofinas.

##### Notes

Known only from Crete and Kos (Gnezdilov et al. 2004). Collected in pitfall traps set from June to September from 639 to 2347 m a.s.l.

#### Mycterodus (Mycterodus) wittmeri

Dlabola, 1974

AACFBE34-228C-54B3-87AB-8F0F6D53625A

https://www.gbif.org/species/4489711

https://hoppers.speciesfile.org/otus/73985

##### Distribution

**Literature data**: Lasithi: Agios Nikolaos (Dlabola 1974b, Nast 1987, Gnezdilov et al. 2014). **New data**: Heraklion: Giouchtas Mt.

##### Notes

Considered endemic to Crete. It was described from Crete and there are no data on its discovery after the initial description. Single specimen from a pitfall trap set in late May and collected in late June at 596 m a.s.l.

#### Semirodus

Dlabola, 1987

A79CDB1C-11C1-5659-8E51-E25F33BD0929

#### * Mycterodus (Semirodus) colossicus

(Dlabola, 1987)

3BC752D5-BAB2-5AEA-AB54-1455B6ACD415

https://www.gbif.org/species/2051149

https://hoppers.speciesfile.org/otus/73987/overview

##### Distribution

**New data**: Heraklion: Koudouma

##### Notes

New species for the fauna of Crete. Known only form Greek islands Rhodes, Karpathos, Naxos and Paros (Gnezdilov and Drosopoulos 2013, Gnezdilov et al. 2014), collected in pitfall traps from June to August up to 812 m a.s.l..

#### Mycterodus (Semirodus) idomeneus

Dlabola, 1984

D5CFEB2D-884F-53DD-8252-7C1FAD1B42FB

https://www.gbif.org/species/2051148

https://hoppers.speciesfile.org/otus/73990

##### Distribution

**Literature data**: Chania: Hora Sfakion (Dlabola 1984) and without specific locations (Gnezdilov et al. 2014). **New data**: Heraklion: Almyros River Park and along the mouth of the Karteros River; Koudouma. Rethymno: Ano Meros; Geropotamos bridge. Chania: Gramvousa Peninsula; Lefka Ori.

##### Notes

Considered endemic to Crete. Apart from the material from the species description, only one male specimen collected in 1975 is mentioned in literature (Gnezdilov et al. 2014). Single specimen from a pitfall trap set in late April and collected in late July at 766 m a.s.l. Collected by sweep netting in May and June up to 412 m a.s.l.

#### Mycterodus (Semirodus) pallens

Stål, 1861

D5039D49-F5D0-51AE-BF0E-24DFCCCEAA99

https://www.gbif.org/species/2051156

https://hoppers.speciesfile.org/otus/73994

##### Distribution

**Literature data**: Chania: Hora Sfakion (Dlabola 1984). **New data**: Chania: Elafonisi. Heraklion: Koudoumas.

##### Notes

Considered endemic to Greece. Collected in pitfall traps from April to October up to 817 m a.s.l.

#### Mycterodus sp.

F35BD76D-1D39-5A63-A891-52E466F0B049

##### Distribution

**New data**: Chania: Kouroupitos; Elafonisi Isl.; Kournas Lake; Gramvousa Peninsula; Lefka Ori mt., Trocharis peak. Rethymno: Lochria; Ano Meros; Rizikas; Kouroutes. Heraklion: Giouchtas Mt.; Koudouma; Anapodaris; Kofinas; Sternes. Lasithi: Pacheia Ammos; Dikti Mt, Limnakaro pl.

##### Notes

All of them are females with a single nymph, collected in pitfall traps left throughout the year up to 1900 m a.s.l.

#### Rhissolepus

Emeljanov, 1971

7B62D892-C687-5DE3-9BC9-8E5118414029

#### Rhissolepus insulanus

(Dlabola, 1982)

B57FDA4D-8CDB-5311-BC4F-BF04E31C561A

https://www.gbif.org/species/5982228

https://hoppers.speciesfile.org/otus/74129/overview

##### Distribution

**Literature data**: Heraklion: Faistos; Matala; Gortys; Amnissos; Anatolien (Dlabola 1982, Gnezdilov et al. 2014, Gnezdilov 2016). **New data**: Heraklion: Keri's forest. Chania: Gramvousa Peninsula.

##### Notes

Known from Greecee and Turkey only. Collected in single pitfall trap from May to June at 142 m a.s.l. and by sweep netting in May at 210 m a.s.l.

#### Tshurtshurnella

Kusnezov, 1927

4C4A7135-9396-53BE-BFEB-795AE92A3C01

#### * Tshurtshurnella pythia

Dlabola, 1979

D05F3D2B-D25D-5BD2-BA11-4ADF1D7CA154

https://www.gbif.org/species/4489646

https://hoppers.speciesfile.org/otus/74288/overview

##### Distribution

**New data**: Chania: Gavdos Isl.; Lefka ori. Heraklion: Koudoumas.

##### Notes

Considered endemic to Greece (Dlabola 1979, Gnezdilov et al. 2014) and new species for the fauna of Crete. Collected in pitfall traps from April to September at 178-2000 m a.s.l.

#### Tettigometridae

Germar, 1821

69D6A691-B583-509C-B0D5-B19A3C2BEAF5

#### Tettigometrini

Germar, 1821

5C8A8353-32E5-58DA-9D76-2D787C9FA13A

#### Tettigometra

Latreille, 1804

BE620965-16F7-5203-BC19-9BEA99BD36AC

#### Tettigometra

Latreille, 1804

115BF347-58DA-5547-8392-58B975B1E131

#### * Tettigometra (Tettigometra) impressifrons

Mulsant & Rey, 1855

4B062B0B-12AE-53F3-9D3C-BE7A48552A6E

https://www.gbif.org/species/2065046

https://hoppers.speciesfile.org/otus/76954

##### Distribution

**New data**: Chania: Gavdos Isl.

##### Notes

New species for the fauna of Crete. This species is widespread in the Mediterranean. Single specimen from a pitfall trap, set in March and collected in June in a wetland on Gavdos Isl. at 259 m a.s.l.

#### ** Tettigometra (Tettigometra) picta

Fieber, 1865

977E634D-D6B0-5678-BFE8-8FAA003674DB

https://www.gbif.org/species/2065034

https://hoppers.speciesfile.org/otus/76962

##### Distribution

**New data**: Chania: Lake Kournas. Heraklion: Koudoumas; Petrokefali; Aposelemis sand dunes; Gazi; Karteros River estuary. Lasithi: between Itanos and Vai.

##### Notes

New species for the fauna of Crete and new for Greece. The species is widespread in the western and central Mediterranean and is common on the islands. Found in pitfall traps from April to December up to 814 m a.s.l. Collected by sweep netting in May and June up to 307 m a.s.l.

#### Tettigometra sp.

5F5E6635-9514-5EA6-8658-BBA2C5032AD8

##### Distribution

**New data**: Rethymno: Prines, *Quercus macrocephalis* forest; Psiloritis, road from Anogia. Agios Mamas; Tigania. Heraklion: Koudouma; Lentas; Sternes; Kofinas.

##### Notes

Collected in pitfall traps from May to November from 69 to 1191 m a.s.l. and by sweep netting in August at 1192 m a.s.l. The specimens are unidentified, as several groups of species of the genus *Tettigometra* in the Mediterranean need to be thoroughly revised before reliable species identification can be made.

#### Delphacoidea

Leach, 1815

C6E2B654-5313-5750-90E9-D71DEFB7157A

#### Cixiidae

Spinola, 1839

9367828F-5F04-578B-B5A6-4E525F63CF9B

#### Cixiinae

Spinola, 1839

8793EFCC-A37B-5833-937D-D4F1C2F920C3

#### Cixiini

Spinola, 1839

30AF2A72-F12B-5804-838B-A72487FFFB06

#### Cixius

Latreille, 1804

D3CBB6A5-E33F-52E8-89C8-E79847F3D2D1

#### Ceratocixius

Wagner, 1939

B8F25986-667B-5724-B92D-7038512BD602

#### * Cixius (Ceratocixius) wagneri

China, 1942

552D4DDD-ECAF-55E6-AB5F-BAFE28DA91CB

https://www.gbif.org/species/2059302

https://hoppers.speciesfile.org/otus/58231/overview

##### Distribution

**New data**: Heraklion: Omalos Plateau at Viannos wetland; Feneromeni wetland; Almyros River.

##### Notes

New species for the fauna of Crete. The species is widespread in southern Europe and parts of the Middle East (Dimitriev et al. 2022). Collected in pitfall traps from March to December at 19-1343 m a.s.l.

#### Tachycixius

Wagner, 1939

A79F6063-10BE-51E9-8D0D-D098FB374507

#### Tachycixius

Wagner, 1939

3341EE0E-4CC7-5E4F-80BF-B047B2F2B72B

#### Tachycixius (Tachycixius) creticus

Dlabola, 1974

9AA4E873-0408-501A-A42B-8821EA7897CE

https://www.gbif.org/species/4489832

https://hoppers.speciesfile.org/otus/58749

##### Distribution

**Literature data**: no specific location (Nast 1987) Chania: Omalos; Sfakia. Rethymno: Gonia. Heraklion: Anogia. Lassithi: Kritsa; Mattia (Dlabola 1974a). **New data**: Chania: Lefka Ori, Troharis peak; road from Anopoli to Pachnes peak. Rethymno: Agios Mamas; Psiloritis; Lohria. Heraklion: Giouchtas Mt.; Kofinas; Koudoumas. Lassithi: Topleni monastery.

##### Notes

Known from Greece and Turkey only (Dlabola 1974a, Dlabola 1987). Collected in pitfall traps from March to September up to 2347 m a.s.l.

#### Pentastirini

Emeljanov, 1971

AC9B2395-1C6F-5D36-9717-16B5058BF81B

#### Hyalesthes

Signoret, 1865

E611CB48-3458-5AFF-925D-57F22222DE27

#### Hyalesthes

Signoret, 1865

990A150F-C72E-5151-96C2-301D931790A6

#### Hyalesthes (Hyalesthes) obsoleta

Signoret, 1865

2FA68028-D303-5446-BD87-0F6B99FABCCA

https://www.gbif.org/species/5157391

https://hoppers.speciesfile.org/otus/59674

##### Distribution

**Literature data**: Chania: Chania (Tsagkarakis et al. 2018) west of Paleochora (Dlabola 1977, Dlabola 1994). Rethymno: north of Moni Preveli (Dlabola 1977). Lasithi: Agios Nikolaos (Dlabola 1977). **New data**: Chania: Gramvousa peninsula; Lake Kournas; Heraklion: near the River Almyros.

##### Notes

Collected in pitfall traps from June to August up to 142 m a.s.l. Collected by sweep netting from May to July on *Vitex* sp. up to 210 m a.s.l.

#### Hyalesthes (Hyalesthes) cf. obsoleta

089B58F7-2219-565A-974F-F9F1DD512EEC

#### Pentastiridius

Kirschbaum, 1868

D63E352D-19D2-5454-9E76-C4E510B857C0

#### Pentastiridius

Kirschbaum, 1868

412F7988-BDB7-5170-A542-61E0B3A96DC7

#### Pentastiridius (Pentastiridius) leporinus

(Linnaeus, 1761)

CDC9C6B1-50A9-57B2-988D-9781C568017E

https://www.gbif.org/species/4489819

https://hoppers.speciesfile.org/otus/60365/overview

Oliarus leporinus (Linnaeus, 1761) in Dlabola (1977)

##### Distribution

**Literature data**: Chania (Dlabola 1977)**. New data**: 20 Rethymno: Lavris, Geropotamos Estuary. Heraklion: Almyros spring.

##### Notes

Single specimen collected from a pitfall trap from June to August at 5 m a.s.l. and three specimens by sweep netting from late May and early June at 4-142 m a.s.l.

#### ** Pentastiridius (Pentastiridius) cf. ovatus

(Metcalf, 1955)

D3B9E533-676A-50B1-A085-1040B7946E7C

https://www.gbif.org/species/5983436

https://hoppers.speciesfile.org/otus/60521/overview

##### Distribution

**New data**: Heraklion: Aposelemis sand dunes

##### Notes

New species for the fauna of Greece. So far, the species is known from Central Asia (Emeljanov 2015). Single specimen collected in a pitfall trap from August to November at 1 m a.s.l.

#### Pentastiridius sp.

4097BB5E-49EA-50FE-8BFE-934FE885ABA6

##### Distribution

**New data**: Heraklion: Aposelemis wetland

##### Notes

Collected in pitfall trap from late July to late November at 1 m a.s.l.

#### Pentastira

Kirschbaum, 1868

62E0AC0F-642B-5C26-A153-74F214CC8628

#### Pentastira cf. demaculata

Dlabola, 1989

1C0B1CF6-7373-5BE4-9EC7-12516BAAC6DA

https://www.gbif.org/species/5157410

https://hoppers.speciesfile.org/otus/60329/overview

##### Distribution

**Literature data**: Chania: Moustakos (Dlabola 1989)**. New data**: Rethymno: Rizikas. Heraklion: opposite site of NHMC exhib; Robos pilot orchard SW.

##### Notes

Considered endemic to Crete. Collected in pitfall traps from June to October at 14-229 m a.s.l.

#### Pentastira demaculata/major

7323310C-1262-5E83-AA8D-9DEE5B335C45

##### Distribution

**New data**: Rethymno: Rizikas; Agios Mamas

##### Notes

Collected in pitfall traps from late April to late July at 251-671 m a.s.l. The specimens show intermediate characteristics between the two species. For their reliable identification, additional materials and methods are needed.

#### Reptalus

Emeljanov, 1971

F0BAAFC2-C1C6-5F01-94F9-4362AF6D988F

#### Pererepa

Emeljanov, 2020

846BEFCE-7372-525A-8C95-E32305E4D8CA

#### Reptalus (Pererepa) panzeri

(Löw, 1883)

1FE64D4C-B1F1-518C-8AF1-DDA1F92C5CF0

https://www.gbif.org/species/2059793

https://hoppers.speciesfile.org/otus/60461/overview

Oliarus panzeri (Löw, 1883) in Dlabola (1977).

##### Distribution

**Literature data**: Rethymno: Dariviana (Dlabola 1977). Heraklion: Moni Preveli; Faistos (Dlabola 1977). **New data**: Chania: Lefka Ori, below Pachnes peak.

##### Notes

Collected by sweep netting in July at 1792 m a.s.l.

#### Reptalus

Emeljanov, 1971

66A25185-FE7C-5686-9DF0-CD72EAAE0259

#### Reptalus (Reptalus) quinquecostatus

(Dufour, 1833)

60A36FD4-5F5C-5647-85DF-C769CC23A268

https://www.gbif.org/species/2059789

https://hoppers.speciesfile.org/otus/60466/overview

Oliarus melanochaetus Fieber, 1876 in Dlabola (1977).

##### Distribution

**Literature data**: Chania (Dlabola 1977). **New data**: Chania: Gramvousa Peninsula. Heraklion: Kofinas (sparse phrygana, sxistolithos); Koudoma-maquis.

##### Notes

Collected in pitfall traps from late June to mid September and in a single trap left throughout the year at 142-813 m a.s.l. The species was recorded for a number of European countries under the name *R. melanochaetus* (Fieber, 1876). However, subsequent studies of the type materials by Webb et al. (2013) have shown that this is a junior synonym of *R. quinquecostatus*. The name *R. quinquecostatus* has been utilised for a considerable period to denote a group of cryptic species (Emeljanov 2020, Tishechkin and Emeljanov 2024).

#### Reptalus sp.

BC744C3B-88AD-5E6F-A133-4282F4DA4447

##### Distribution

**New data**: Chania: Gramvousa Peninsula. Rethymno: Moni Preveli. Heraklion: Kofinas; Koudoumas.

##### Notes

Collected from pitfall traps from April to September from 142 to 786 m a.s.l. and from a single trap left throughout the year at 813 m a.s.l.

#### Delphacidae

Leach, 1815

728CFF85-988F-59DB-8193-1A50783A216B

#### Asiracinae

Motschulsky, 1863

44F5226B-AC9D-569A-8419-B557CB43C373

#### Asiracini

Motschulsky, 1863

301A2A19-828C-572C-B346-380BB2D7F708

#### Asiraca

Latreille, 1797

3775DA1C-1169-5ABA-8354-E606CD5B6B5A

#### Asiraca clavicornis

(Fabricius, 1794)

A6C52030-5ADE-5EF9-878D-954FE95590BD

https://www.gbif.org/species/2054367

https://hoppers.speciesfile.org/otus/60903

##### Distribution

**Literature data**: Rethymno: Vizari; Agia Galini; Spil; Dariviana; Anguselliana. Heraklion: Gortis; Tympaki (Asche and Remane 1982). **New data**: Heraklion: near the Almyros River.

##### Notes

Collected in a pitfall trap set in late November and collected in late May near a river at 5 m a.s.l.

#### Delphacinae

Leach, 1815

729FDAF5-3DD7-5C41-ADEA-A8113C9D8E74

#### Delphacini

Leach, 1815

2F652F86-AE04-5142-91AE-1FEE1CF3339D

#### Delphacina

Leach, 1815

15E6CCD1-7B32-5177-8772-977200533D8C

#### Chloriona

Fieber, 1866

01B19A8E-36A8-5DE7-BB58-7DD3D3515215

#### Chloriona unicolor

(Herrich-Schäffer, 1835)

C5206A41-BAFC-56B3-8B7F-B296B44AB7B4

https://www.gbif.org/species/2054413

https://hoppers.speciesfile.org/otus/61276

##### Distribution

**Literature data**: Rethymno: Agia Galini; Dariviana. Heraklion: Matala; Tympaki; Azimion (Asche 1982, Asche and Remane 1982). **New data**: Heraklion: Analipsi.

##### Notes

Collected by sweep netting on various herbaceous vegetation under olive groves in early July at 15 m a.s.l.

#### Chloriona sp.

B1F04BB4-9737-5D5B-9C3C-D67C290C7E0F

##### Distribution

**New data**: Rethymno: Lavris. Heraklion: Nea Alikarnassos.

##### Notes

Two females collected by sweep net in June by the riverside.

#### Eurysa

Fieber, 1866

1A6739E4-7540-5118-BD2D-0CB47463B2C4

#### Eurysa

Fieber, 1866

ED92548A-7557-5131-9161-2BF920A1558C

#### Eurysa (Eurysa) duffelsi

Drosopoulos & Asche, 1984

62B9D0AA-85F8-50AA-81B3-8A2AB5819281

https://www.gbif.org/species/2056911

https://hoppers.speciesfile.org/otus/61787

##### Distribution

**Literature data**: Chania: Samaria Gorge. Rethymno: Moni Arcadio; between Perama and Heraklion (Drosopoulos and Asche 1984). **New data**: Heraklion: Kofinas.

##### Notes

In the higher parts of the Samaria Gorge on grasses under trees of the genera Pinus and *Abies cephalonica* (Drosopoulos and Asche 1984).

#### * Eurysa (Eurysa) lineata

(Perris, 1857)

BAF4C5D7-DAB0-5A7A-94A9-F5F04834F28C

https://www.gbif.org/species/2056935

https://hoppers.speciesfile.org/otus/61802

##### Distribution

**New data**: Heraklion: Koudoumas.

##### Notes

New species for the fauna of Crete. The species is described from Tunisia and widespread in southern Europe and parts of the Middle East and Central Asia (Dimitriev et al. 2022). Caught in pitfall trap set in late December and collected in late January at 761 m a.s.l.

#### Eurysa sp.

0C336575-90EF-5D29-AA2E-AFE0CD7FC473

##### Distribution

**New data**: Heraklion: Omalos Vianou.

##### Notes

Single female specimen, collected by pitfall trap fom May to July 1343 m a.s.l.

#### Muirodelphax

Wagner, 1963

1020CC65-46F9-5F39-9597-7342D0296FD6

#### * Muirodelphax aubei

(Perris, 1857)

0A71510A-A7BE-5B09-B12C-E46FC1376F3B

https://www.gbif.org/species/2053946

https://hoppers.speciesfile.org/otus/62582

##### Distribution

**New data**: Chania: Stavros; Lefka Ori on the road to Pachnes peak. Heraklion: Karteros Estuary; on the dunes of Aposelemis Beach.

##### Notes

New species for the fauna of Crete. The species is widespread in the temperate zones of the Palaearctic (Dimitriev et al. 2022). Collected in pitfall traps and by sweep netting from May to July up to 1792 m a.s.l.

#### Perkinsiella

Kirkaldy, 1903

5FF63734-82C0-5E6F-AD30-AFBB723B1F23

#### * Perkinsiella sp.

762558D4-DB1C-505C-8A2B-C44C3BC65389

##### Distribution

**New data**: Chania: Falasarna wetland.

##### Notes

New genus for Crete. Single specimen collected from a pitfall trap in May at 2 m a.s.l. Identification of the species is currently impossible, as we only have one female specimen.

#### Pseudaraeopus

Kirkaldy, 1904

220C8CD3-23F3-56ED-B06A-CA075BD09009

#### Pseudaraeopus lethierryi

(Mulsant & Rey, 1879)

A961D4EC-C6F5-5B6C-B727-55BD59BC8055

https://www.gbif.org/species/2056380

https://hoppers.speciesfile.org/otus/63118/overview

##### Distribution

**Literature data**: Rethymno: Melambes Vill. (Asche and Remane 1982). **New data**: Rethymno: Geropotamos Bridge.

##### Notes

Collected by sweep netting in the beginning of June at 32 m a.s.l.

#### Toya

Distant, 1906

7994E13F-D19A-5C10-99A9-358AD35C088A

#### Metadelphax

Wagner, 1963

D987B2A6-B8CA-5A41-8AC8-B8956D598606

#### Toya (Metadelphax) propinqua

(Fieber, 1866)

2AF481EA-68B5-5033-BA5B-D771932748EB

https://www.gbif.org/species/9367508

https://hoppers.speciesfile.org/otus/62481

##### Distribution

**Literature data**: Rethymno: Agia Galini; Apodoulou; Meronas. Heraklion: Psiloritis (Asche and Remane 1982). **New data**: Chania: Elafonisi; Falasarna. Heraklion: Kofinas; near the Almyros River.

##### Notes

Collected in pitfall traps from March to November up to 786 m a.s.l.

#### Tropidocephalini

Muir, 1915

05F240E2-E2BF-5BF8-91CB-756735676EB0

#### Tropidocephala

Stål, 1853

DA655B5C-87C1-50CA-A282-E03BBF8FA624

#### Tropidocephala tuberipennis

(Mulsant & Rey, 1855)

91D89FEB-F472-5EC3-BE55-D897F6D8DECA

https://www.gbif.org/species/2056170

https://hoppers.speciesfile.org/otus/64081

##### Distribution

**Literature data**: Rethymno: Agia Galini; Psiloritis. Heraklion: Timbaki (Asche and Remane 1982)**. New data**: Rethymno: Lavris.

##### Notes

Collected in early June by sweep netting on *Imperata* sp. near Geropotamos River, 5 m a.s.l.

#### Kelisiinae

Wagner, 1963

84389730-9403-5619-AF5D-924BCBE715DF

#### Kelisia

Fieber, 1866

0A25C999-CD78-5F2C-95B2-71904BC538D9

#### Kelisia creticola

Asche, 1982

8A96B6CD-DA59-523C-9510-CFE853018E20

https://www.gbif.org/species/2056817

https://hoppers.speciesfile.org/otus/64249

##### Distribution

**Literature data**: Rethymno: Kares; Psiloritis. Heraklion: Spili Galia; Zaros (Asche 1982). **New data**: Chania: Falasarna; Agia Irini Gorge on E4 path.

##### Notes

Considered endemic to Crete. Collected from July to November in pitfall traps in a wetland and by sweep netting in early June on *Juncus* sp., in Agia Irini Gorge at 553 m a.s.l.

#### Kelisia sp.

EA7B8B87-786C-55F8-B51C-BBFA9F2A694A

##### Distribution

**New data**: Chania: Falasarna wetland; Agia Irini george, the E4 path. Heraklion: Almyros River.

##### Notes

Collected in pitfall traps from June to November at 2 m a.s.l. and by sweep netting in June at 553 m a.s.l. Female specimens that we cannot identify by the colouration of their heads.

#### Cicadomorpha

Evans, 1946

5FD68A01-4FB0-5D9D-BEBB-B67B7E64EA07

#### Cicadoidea

Latreille, 1802

645C5FF0-BFB3-5AC4-BAC8-44D2041F7DD5

#### Cicadidae

Latreille, 1802

2C6EB610-9936-52A2-BF26-DF05CF7C34B9

#### Cicada

Linnaeus, 1758

3286ABA2-A856-5BA6-9250-BED725B2BD79

#### Cicada cretensis

Quartau & Simões, 2005

C97EC962-F66A-512B-8321-44C9B2AE420B

https://www.gbif.org/species/8056491

https://hoppers.speciesfile.org/otus/8759

##### Distribution

**Literature data**: Chania: Omalos. Rethymno: Akoumia; Spili. Heraklion: Pyrgos; Tilisos. Lasithi: Ierapetra; Pilalimata; Vai (Quartau and Simões 2005). **New data**: Chania: Gavdos Isl.; Anopoli and Pachnes in Lefka Ori; archaeological site Aptera; Falasarna. Rethymno: Nida plateau in Psiloritis. Heraklion: Alagni; Giouchtas Mt.; Platanos; Mandres; Siva; between Peri and Platanos; Rouvas; Petrokefali; Trapsano; Moni Vrontisi; Gazi; NHMC yard; Analipsi.

##### Notes

Known only from Crete, Karpathos and Kythira islands (Simões and Quartau 2008).

#### Oligoglena

Horváth, 1912

99DAE243-CEEA-569D-9E2C-04D8C497F3C2

#### Oligoglena carayoni

(Boulard, 1982)

1BF0A344-7100-5DF5-8C4B-E312CCFD8D97

https://www.gbif.org/species/9612587

https://hoppers.speciesfile.org/otus/7215

Tettigetta carayoni Boulard 1982 in Trilar and Gogala (2015).

##### Distribution

**Literature data**: Rethymno: Moni Arkadi (Trilar and Gogala 2015). **New data**: Chania: Therissos Gorge. Rethymno: Prines. Heraklion: Petrokephali; Archanes; Analipsi. Lasithi: Xirokampos.

##### Notes

Considered endemic to Crete. Collected in pitfall traps from April to August and by sweep netting in July up to 435 m a.s.l.

#### Pagiphora

Horvath, 1912

2F5E8E6E-C25D-5EB4-83E1-7182BE329980

#### Pagiphora aschei

Kartal, 1978

852E8B62-55A6-5F98-A77D-7BE36E428CB7

https://www.gbif.org/species/4482657

https://hoppers.speciesfile.org/otus/8005

##### Distribution

**Literature data**: Chania: Gramvousa Peninsula; Balos; Kissamos. Rethymno: Agia Galini; Adele; Armeni; Platanias; Spili. Heraklion: Ammoudara Beach; Knossos. Lasithi: Agios Nikolaos; Neapoli (Trilar and Gogala 2012). **New data**: Heraklion: Petrokefali.

##### Notes

Collected in pitfall traps are from May to July at 45 m a.s.l.

#### Cercopoidea

Leach, 1815

1A856D42-62EF-5868-A9D7-C9F971A4878B

#### Aphrophoridae

Amyot & Audinet-Serville, 1843

CB34BF7F-515A-5C51-BC3B-893A0CE47353

#### Lepyronia

Amyot & Serville, 1843

E4AB8AA4-C21D-55D9-8BBB-8590D473BB92

#### * Lepyronia coleoptrata

(Linnaeus, 1758)

C4A633D5-1024-5A18-B677-FA5C76E033B3

https://www.gbif.org/species/2015789

https://hoppers.speciesfile.org/otus/1044335

##### Distribution

**New data**: Heraklion: along the Almyros River.

##### Notes

New genus and species for the fauna of the Island of Crete. The species is widespread in the temperate zones of the Palaearctic (Dimitriev et al. 2022). Collected in pitfall traps from May to August at 5-20 m a.s.l.

#### Neophilaenus

Haupt, 1935

5819EC61-6230-56E8-B219-12DCEC594215

#### Neophilaenus

Emeljanov, 1964

53A5D734-3C71-5E87-B9B2-3BE1B6D08044

#### Neophilaenus (Neophilaenus) campestris

(Fallén, 1805)

454A60EA-AB55-5362-B161-DBFCE7979D02

https://www.gbif.org/species/4482781

https://hoppers.speciesfile.org/otus/1660

##### Distribution

**Literature data**: Chania (Koufakis et al. 2024). **New data**: Heraklion: Almyros River Park; Giouchtas Mt.; Chania: Gavdos Isl.

##### Notes

Collected in pitfall traps from March to July and by sweep netting in May on various herbaceous vegetation at 30-1193 m a.s.l.

#### Philaenus

Stål, 1864

9A03F126-F09B-5ADE-95FF-AB89343F0768

#### Philaenus signatus

Melichar, 1896

ED3EA1DB-66E1-5CFE-AA87-A102982E5B95

https://www.gbif.org/species/2016065

https://hoppers.speciesfile.org/otus/1800

##### Distribution

**Literature data**: Chania: no specific location. Heraklion: no specific location (Drosopoulos and Asche 2000). **New data**: Chania: Gramvousa Peninsula. Rethymno: Exantis; Moni Preveli. Heraklion: Almyros River Park; Giouchtas Mt.; Mohos surrounding.

##### Notes

Colour polymorphism is observed. In Greece, it usually lives in the same habitat as *Philaenus spumarius*, more often near the sea, but there are also single finds from the mountains (Drosopoulos and Asche 2000). Caught in pitfall traps left throughout the year and by sweep netting - in May and July on various herbaceous plants up to 800 m a.s.l.

#### Philaenus spumarius

(Linnaeus, 1758)

F902C4CA-BF73-5E2F-9875-C2E9D2A6A1A3

https://www.gbif.org/species/2016038

https://hoppers.speciesfile.org/otus/1804

##### Distribution

**Literature data**: no specific locality (Nast 1972) Chania: Chaleppa; Governeto (Haupt 1928); Chania (Tsagkarakis et al. 2018); Heraklion: no specific location (Drosopoulos and Asche 2000). **New data**: Chania: Kournas lake. Rethymno: Prines; Preveli. Heraklion: Park on the Almyros River; Analipsi along the Aposelemi River; Mochos surrounding, Omalos Plateau. Lassithi: Lassithi Plateau; Selakano.

##### Notes

Colour polymorphism is observed. Caught in pitfall traps left throughout the year and through sweep netting - in May and July on various herbaceous plants up to 1334 m a.s.l.

#### Cercopidae

Leach, 1815

72D42B6B-0C61-5A4A-AB72-0BCB9B01A6E6

#### Cercopis

Fabricius, 1775

45375758-356D-5DC5-9FA6-D66F3FB635AE

#### * Cercopis sanguinolenta

(Scopoli, 1763)

47819CAA-FE1E-5C7C-9D42-1753969BBAB6

https://www.gbif.org/species/2018539

https://hoppers.speciesfile.org/otus/2530

##### Distribution

**New data**: Heraklion: along the Almyros River.

##### Notes

New genus and new species for the fauna of Crete. The species is widespread in Europe and parts of the Middle East (Dimitriev et al. 2022). Single specimen collected in a pitfall trap set from November to May at 5 m a.s.l.

#### Haematoloma

Haupt, 1919

311C16FB-CD10-5DB0-984C-8171FE995F56

#### * Haematoloma dorsatum

(Ahrens, 1812)

3A54923B-6953-5412-8378-0C673A737CCF

https://www.gbif.org/species/2017811

https://hoppers.speciesfile.org/otus/2567

##### Distribution

**New data**: Heraklion: Moni Koudouma.

##### Notes

New genus and new species for the fauna of Crete. The species is widespread in the Mediterraneum (Dimitriev et al. 2022). Collected in pitfall traps in April-May at 523-598 m a.s.l.

#### Membracoidea

Rafinesque, 1815

3252D70E-3A2B-53DA-B3E8-0E993A0A402E

#### Cicadellidae

Latreille, 1802

AB1AA87F-0DAE-5FA3-ADA1-182E16FC29AD

#### Aphrodinae

Haupt, 1927

42AFFFA3-2142-52D6-A275-24C37975D8B0

#### Aphrodini

Haupt, 1927 [1859]

27F7288B-CF5F-5153-8E63-43CB96B60174

#### Anoscopus

Kirschbaum, 1858

3C882C23-7F29-5540-91AB-77B2491E31B7

#### Anoscopus albifrons

(Linnaeus, 1758)

8E8A7CEC-F1C4-58F9-8799-BF86816928F0

https://www.gbif.org/species/4484088

https://hoppers.speciesfile.org/otus/13604

Aphrodes albifrons (Linnaeus, 1758) in Dlabola (1977), Lymberakis (2003).
Aphroderes albifrons L. Haupt (1928).

##### Distribution

**Literature data**: no specific locations (Dlabola 1977), Chania: Lefka Ori (Lymberakis 2003); Meskla (Haupt 1928). **New data**: Chania: Gramvousa Peninsula; Agia Lake; Falasarna; Elafonisi; Lefka Ori; Kournas Lake. Rethymno: Petres; Moni Preveli; Vrises; Agia Fotini; Platania. Heraklion: Aposelemis Dam; Partiron Dam; Faneromeni Dam; along the Almyros River; Siva; Rufas; Voroi; Koudouma; Kofinas; Giouchtas Mt.; Omalos plateau; Psiloritis, Vrontisi monastery; Lasithi: Dikti Mt.; Pachia Amos; Krustas; Istro; Tripti M; Agios Nikolaos; between Itanos and Vai.

##### Notes

The species has been collected in pitfall traps left throughout the year up to 1700 m above sea level.

#### * Anoscopus flavostriatus

(Donovan, 1799)

78E1F5F2-E5FE-5291-8092-0C2CEE05E5C3

https://www.gbif.org/species/4484084

https://hoppers.speciesfile.org/otus/13699

##### Distribution

**New data**: Chania: Lake Agia.

##### Notes

New species for the fauna of Crete. The species is widespread in Holarctic realm (Dimitriev et al. 2022). Collected in pitfall traps set during the period May – July at 44 m a.s.l. Epigeobiont.

#### ** Anoscopus gorloppus

Guglielmino & Bückle, 2015

D74D8237-5C70-5E97-8591-D7CD2FBBA011

https://www.gbif.org/species/11174939

https://hoppers.speciesfile.org/otus/13709

##### Distribution

**New data**: Heraklion: along the Almyros River.

##### Notes

New species for the fauna of Greece. So far, the species is known only from Italy, where it was described (Guglielmino and Bückle 2015). Single specimen, collected in a pitfall trap between June and August at 1 m a.s.l. Epigeobiont.

#### ** Anoscopus samuricus

(Tshmir, 1977)

4BEAA421-DDD4-5056-88B4-FDE862256C70

https://www.gbif.org/species/4484083

https://hoppers.speciesfile.org/otus/13720

##### Distribution

**New data**: Rethymno: Moni Preveli; Psiloritis Mt. Heraklion: Kofinas; Omalos plateau.

##### Notes

New species for the fauna of Greece. The species is known from Dagestan, the north-western Caucasus, Iraq, Turkey and Spain (Guglielmino and Bückle 2015, Özgen et al. 2024). Collected in pitfall traps from March to July at 20-1334 m a.s.l.

#### Aphrodes

Curtis, 1831

49B67379-541D-55DB-9159-E209BC093D92

#### Aphrodes bicincta

(Schrank, 1776)

CAEC340B-EF53-5AFC-B5E4-98FCD60E5517

https://www.gbif.org/species/4484106

https://hoppers.speciesfile.org/otus/13756

##### Distribution

**Literature data**: no specific locality (Nast 1972). Chania: no specific location (Abdollahi et al. 2015, Tsagkarakis et al. 2018); Lefka Ori (Lymberakis 2003); Kydonia (Haupt 1928). **New data**: Chania: Troharis peak in Lefka Ori; Lake Kournas. Rethymno: Sakturia; Psiloritis. Heraklion: Kofinas.

##### Notes

Collected in traps from April to September up to 2347 m a.s.l.

#### ** Aphrodes diminuta

Ribaut, 1952

C228301F-E04F-5FC5-A9C8-0621924F726B

https://www.gbif.org/species/7508026

https://hoppers.speciesfile.org/otus/13841

##### Distribution

**New data**: Chania: Troharis peak in Lefka Ori.

##### Notes

New species for the fauna of Greece. The species is widespread in the temperate Palaearctic realm (Dimitriev et al. 2022). Two specimens from pitfall traps in September under Troharis peak at 2347 m a.s.l.

#### Aphrodes makarovi

Zachvatkin, 1948

06B95206-F841-513B-AA11-31A28C55E8AA

https://www.gbif.org/species/4484105

https://hoppers.speciesfile.org/otus/13847

##### Distribution

**Literature data**: Chania: no specific location (Antonatos et al. 2020). **New data**: Chania: Troharis peak and on the road to Pachnes peak in Lefka Ori. Rethymno: Moni Preveli.

##### Notes

Collected in pitfall traps from March to October up to 2347 m a.s.l.

#### Aphrodes sp.

EF47D4B4-24FA-54B6-8A21-1CE25EC89F26

##### Distribution

**New data**: Chania: Anopoli, phrygana plateau; Therisso to Kaloros Mt. Rethymno: Moni Preveli ; Agios Mamas; Tigania. Heraklion: Kofinas; Achentrias; Mikri Episkopi.

##### Notes

Nine females collected in pitfall traps left throughout the year up to 1780 m a.s.l.

#### Deltocephalinae

Fieber, 1869

A89E4157-4892-5D9D-9341-49BB65472178

#### Athysanini

Van Duzee, 1892

728485D4-D73D-5082-B42D-B9DB7E9DC43B

#### Allygidius

Ribaut, 1948

4C70E3FE-5048-504C-9462-E0616804D9D9

#### Allygidius sp.

FD4E8879-F1BA-5B48-824B-81928677017A

##### Distribution

**New data**: Heraklion: Almyros River; Kommos sand dunes.

##### Notes

Five females, collected in pitfall traps from November to May.

#### Allygus

Fieber, 1872

95654685-053F-5A55-B6E5-ED9F339E0056

#### * Allygus modestus

Scott, 1876

4C5A7510-89AC-5D2C-80DC-FDB76F6BFF9C

https://www.gbif.org/species/2029993

https://hoppers.speciesfile.org/otus/20431

##### Distribution

**New data**: Heraklion: Giouchtas Mt.

##### Notes

New species for the fauna of the Crete. The species is known from Europe and North Africa, but is registered as non-indigenous in North America (Hamilton 1983, Dimitriev et al. 2022). Collected in May by sweep netting at 700 m a.s.l. on shrubby vegetation.

#### Allygus sp.

82ED1598-471A-536E-A114-D0852444326B

##### Distribution

**New data**: Heraklion: Almyros River; Livadopa pilot orchard, Siva.

##### Notes

Two females collected in pitfall traps in May.

#### Conosanus

Osborn & Ball, 1902

FC0BEDD3-7FB0-5B34-AADC-B4A6F0CF3647

#### Conosanus obsoletus

(Kirschbaum, 1858)

5699CE8A-9986-56C6-A494-7925B85F0197

https://www.gbif.org/species/2045459

https://hoppers.speciesfile.org/otus/20883

##### Distribution

**Literature data**: Chania: Chania (Tsagkarakis et al. 2018). **New data**: Heraklion: along the Aposelemis River.

##### Notes

Collected in pitfall traps from May to August up to 5 m a.s.l.

#### Euscelidius

Ribaut, 1942

12CB7DB1-5E59-5333-B8A9-3D798EE68A29

#### Euscelidius variegatus

(Kirschbaum, 1858)

27F128DA-4BB8-57A4-925C-A3B83222B2F6

https://www.gbif.org/species/2026086

https://hoppers.speciesfile.org/otus/21028

##### Distribution

**Literature data**: Chania: Zimbragos; Nerokouros; Pyrgos; Louzaki; Zonaki; Souda. Rethymno: Episkopi. Heraklion: Fodele (Koufakis et al. 2022). **New data**: Heraklion: along the Aposelemis River.

##### Notes

Collected in pitfall traps from May to August up to 20 m a.s.l.

#### Euscelis

Brullé, 1832

8D7E562C-8DCE-5ECE-84DF-E75D7FB653F3

#### * Euscelis incisa

(Kirschbaum, 1858)

DF8F08A5-8BFC-59E0-BECD-F543DED51D31

https://www.gbif.org/species/8929628

https://hoppers.speciesfile.org/otus/21077

##### Distribution

**New data**: Heraklion: along the Aposelemis River; Karteros; Kamilari; Platanos.

##### Notes

New species for the fauna of Crete. Widespread Palaearctic species (Dimitriev et al. 2022). Collected in pitfall traps from May to August up to 93 m a.s.l. and by sweep netting in June on dry grasses near the sea coast.

#### Euscelis ohausi

Wagner, 1939

AAE60703-8311-5932-A515-15881283B5F4

https://www.gbif.org/species/5154903

https://hoppers.speciesfile.org/otus/21104

##### Distribution

**Literature data**: Chania: Zimbragos; Nerokouros; Pyrgos; Louzaki; Zunaki; Souda. Rethymno: Episkopi. Heraklion: Fodele; Agia Varvara (Koufakis et al. 2022). **New data**: Rethymno: Moni Preveli; Rizikas. Heraklion: Siva; Mikri; Episkopi; Feneromeni; near the Almyros River; Temenos; Agios Spyridon; Peri; Platanos; Amoudara. Lasithi: Mochlos.

##### Notes

Collected in pitfall traps set from April to December up to 315 m a.s.l.

#### Euscelis

Brullé, 1832

249221FD-E18E-5B21-9DC9-19C71E0D2B0B

#### Euscelis (Euscelis) lineolata

Brullé, 1832

8319C714-56B8-55DD-AD6D-ECACCB1E6C40

https://www.gbif.org/species/5154915

https://hoppers.speciesfile.org/otus/21052

##### Distribution

**Literature data**: Chania: no specific location (Tsagkarakis et al. 2018, Antonatos et al. 2020). **New data**: Rethymno: Ano Meros; Psiloritis. Heraklion: Siva, Kofinas. Lassithi: Bramiana Dam.

##### Notes

Collected in pitfall traps left throughout the year up to 729 m a.s.l.

#### Euscelis sp.

23B2EF66-0E2B-503C-9D30-CCF8C9EFC2C1

##### Distribution

**New data**: Chania: Agia Lake; Kouroupitos. Heraklion: Anapodaris; Koudoumas; Sternes; Almyros River; Voroi

##### Notes

Nine females collected by pitfall traps left throughout the year round up to 607 m a.s.l.

#### Melillaia

Linnavuori, 1971

C33239A6-E3BF-57ED-9683-5D6B29B335C0

#### Melillaia desbrochersi

(Lethierry, 1889)

89E293E6-FD9E-5EAF-9863-9680B9B3EE03

https://www.gbif.org/species/2033546

https://hoppers.speciesfile.org/otus/21531

Melillaia matsumuri (Metcalf, 1955) in Nast (1972).

##### Distribution

**Literature data**: no specific location (Nast 1972, de Jong et al. 2014). **New data**: Chania: Gramvousa Peninsula; Elafonisi; Gavdos Isl. Rethymno: Moni Preveli; Amari Dam. Heraklion: Giouchtas Mt.; Koudoumas; Pigidakia; Aposelemys wetland. Lassithi: Mochlos; Topleni monastery.

##### Notes

Collected in pitfall traps left throughout the year up to 770 m a.s.l. and by sweep netting only in May.

#### Platymetopius

Burmeister, 1838

BB8C5F90-359F-5816-88DD-8BDE1B61B7D5

#### Platymetopius sp.

6B4219B8-5980-5D06-BF9D-58DAB814C82B

##### Distribution

**New data**: Heraklion: Giouchtas Mt.

##### Notes

Single nymph collected by sweep net in May at 800 m a.s.l.

#### Thamnotettix

Zetterstedt, 1837

125DFD23-E579-5BDF-9F03-E563BE5CEF19

#### * Thamnotettix zelleri

(Kirschbaum, 1868)

01928F76-B597-59B3-928B-EBC9A447CF67

https://www.gbif.org/species/2026125

https://hoppers.speciesfile.org/otus/22289

##### Distribution

**New data**: Rethymno: Prines. Heraklion: Siva; Petrokephali.

##### Notes

New species for the fauna of Crete. Widespread Mediterranean species (Dimitriev et al. 2022). Collected in pitfall traps from April to May at 41-196 m a.s.l.

#### Thamnotettix minoidis

Dlabola, 1974

1B6C3B47-266A-52B5-A0CF-7ADCDB205CE0

https://www.gbif.org/species/4483120

https://hoppers.speciesfile.org/otus/22249

##### Distribution

**Literature data**: Lasithi: Neapolis (Dlabola 1974a). **New data**: Rethymno: Psiloritis. Heraklion: Giouchtas Mt.; Kofinas.

##### Notes

Considered endemic to Crete. Collected in pitfall traps from February to August up to 1100 m a.s.l.

#### Chiasmini

Distant, 1908

D60E2A23-E0CC-556A-AE4C-9CCEA55CEFDA

#### Aconura

Lethierry, 1876

380C80E5-18F5-5DC3-881D-D70302D0BBF3

#### Aconura

Lethierry, 1876

9945FB9F-5732-549E-8202-EE164706B805

#### ** Aconura (Aconura) jakowlefi

Lethierry, 1876

52B3A9E0-2742-55CF-A3D4-0A1F452B327F

https://www.gbif.org/species/2036594

https://hoppers.speciesfile.org/otus/22824

##### Distribution

**New data**: Chania: Gavdos Isl.

##### Notes

New species for the fauna of Greece. So far, it has been found in areas around the Black Sea, the Caspian Sea and Central Asia (Dimitriev et al. 2022). Caught in pitfall traps left throughout the year – from November to March and from June to August at 30 m a.s.l. One of the southernmost parts of Gavdos Island, which is also the southernmost part of Greece.

#### Chiasmus

Muslant & Rey, 1855

6F2E3EC3-B706-52FD-B987-0DA693979E18

#### * Chiasmus conspurcatus

(Perris, 1857)

33129FB5-E245-55ED-A2C1-443CE1748791

https://www.gbif.org/species/5155552

https://hoppers.speciesfile.org/otus/23088

##### Distribution

Chania: Gavdos Isl.

##### Notes

New genus and species for the fauna of Crete. Widespread in Europe, the Mediterranean and Central Asia (Dimitriev et al. 2022). Collected in pitfall traps from June to August at 259 m a.s.l. on the north-western side of Gavdos Isl.

#### Exitianus

Ball, 1929

E385B56B-5011-5204-8F68-14F0C94D2F55

#### Exitianus africanus

(Walker, 1851)

B1D333C9-50AB-5D98-870C-6BB44E05E5AA

https://www.gbif.org/species/2048928

https://hoppers.speciesfile.org/otus/23200

Exitianus capicola (Stål, 1855) in Dlabola (1977), Tsagkarakis et al. (2018), Antonatos et al. (2020).

##### Distribution

**Literature data**: Chania: Near the Kiliaris River. Heraklion: Platanos; Faistos; Almyros (Dlabola 1977, Tsagkarakis et al. 2018, Antonatos et al. 2020). **New data**: Heraklion: Platanos; near the Almyros River.

##### Notes

Collected in pitfall traps from June to September up to 93 m a.s.l.

#### Cicadulini

Van Duzee, 1892

151D65C2-10CF-5637-BFD4-6FF1BCE7FF37

#### Cicadula

Zetterstedt, 1837

132BC3FF-84D3-5202-8FEB-643D39B0F14A

#### Cicadula sp.

163CFBF4-D44A-57CF-B45F-4EA3D0CD9AD7

##### Distribution

**New data**: Chania: Gramvousa Peninsula. Heraklion: Moires.

##### Notes

Single female specimen collected in a pitfall trap from April to late June at 419 m a.s.l. and, by sweep netting, another single female specimen in late July at 77 m a.s.l.

#### Eohardya

Zakhvatkin, 1946

BAC435DC-A765-521F-9CD9-5875D3272B64

#### Eohardya cf. fraudulenta

(Horváth, 1903)

3BCA6F70-B669-589E-9BFA-80EC5A426057

https://www.gbif.org/species/4483125

https://hoppers.speciesfile.org/otus/21333/overview

##### Distribution

**Literature data**: no specific locality (Dlabola 1994). **New data**: Chania: Pachnes peak in Lefka Ori Mt. Heraklion: Moires; Omalos Vianou in Dikti Mt.; Kofinas; Koudoumas; Sternes.

##### Notes

Collected in pitfall traps from May to December at 523-2453 m a.s.l. and by sweep netting at the end of July at 77 m a.s.l.

#### Mocydiopsis

Ribaut, 1939

1A1DFB96-06BF-5AF4-87F1-4BE298221D6B

#### Mocydiopsis sp.

4837E404-FBC3-5BBF-B7DC-B267BCD378C6

##### Distribution

**New data**: Rethymno: Afentis Christos. Heraklion: Koudouma; Kofinas.

##### Notes

New genus for the fauna of Crete. Collected in pitfall traps from June to August up to 729 m a.s.l.

#### Proceps

Mulsant & Rey, 1855

B96FBF76-9449-57D1-95D2-637545A2C7F2

#### * Proceps acicularis

Mulsant & Rey, 1856

5DBE746F-09EB-500C-A019-937D9419ACDE

https://www.gbif.org/species/2044330

https://hoppers.speciesfile.org/otus/23602

##### Distribution

**New data**: Rethymno: Moni Preveli. Heraklion: Siva; Kofinas.

##### Notes

New genus and new species for the fauna of Crete. The species is widespread in Europe and Central Asia (Dimitriev et al. 2022). Found in pitfall traps left throughout the year, placed at altitudes from 91 to 175 m a.s.l.

#### Deltocephalini

Fieber, 1869

30EF5F30-C620-5422-828A-43C29A40E417

#### Maiestas

Distant, 1917

FC3230AD-DF63-5051-9551-B1982C28311C

#### Maiestas schmidtgeni

(Wagner, 1939)

13EB7521-2581-5CBC-B62C-E7B638883AF6

https://www.gbif.org/species/7677719

https://hoppers.speciesfile.org/otus/24451

##### Distribution

**Literature data**: Chania: Kyriaris River. Heraklion: Faistos (Dlabola 1977, Tsagkarakis et al. 2018). **New data**: Rethymno: Lavris. Heraklion: near Aposelemis River.

##### Notes

Collected in pitfall traps from April to August and by sweep netting in June. In wetlands and lower riverbeds at low altitudes (up to 4 m).

#### Fieberiellini

Wagner, 1951

6E3CF622-3D35-533F-9AD1-325A00BD9A76

#### Fieberiella

Signoret, 1880

1D3781AD-A9B0-5181-BC8A-A65DB9BAE972

#### Fieberiella kritiella

Dlabola, 1989

DF62B08C-237C-563D-A78D-4BACCDC8176E

https://www.gbif.org/species/2043589

https://hoppers.speciesfile.org/otus/25363

##### Distribution

**Literature data**: Chania: Lake Kournas (Dlabola 1989). **New data**: Chania: Lefka Ori - Troharis peak. Rethymno: Moni Preveli; Psiloritis. Heraklion: Kofinas.

##### Notes

Considered endemic to Crete. Collected in pitfall traps from March to October from 167 to 2347 m a.s.l.

#### Fieberiella sp.

8D089482-E758-56F0-BB1C-04B2FD33999F

##### Distribution

**New data**: Heraklion: NHMC yard.

##### Notes

Single female specimen collected by sweep net in July at 116 m a.s.l.

#### Synophropsis

Haupt, 1926

F650D44E-D957-56DB-A894-BF0D972D1F52

#### Synophropsis lauri

(Horváth, 1897)

84AE69DF-B466-5DEC-8B68-B37755F386F6

https://www.gbif.org/species/2046028

https://hoppers.speciesfile.org/otus/25405

##### Distribution

**Literature data**: Chania no specific locality. Rethymno: Moni Preveli. Lassithi: Agios Ioannis (Dlabola 1977, Tsagkarakis et al. 2018, Antonatos et al. 2020). New data: Heraklion: Roufas. Lassithi: Istro, Xerokampos.

##### Notes

Collected in pitfall traps from May to August up to 299 m a.s.l.

#### Goniagnathini

Wagner, 1951

E11B98BC-3CD0-56BD-AB43-7A42CE078F43

#### Epistagma

Emeljanov, 1999

F41877F3-5098-5D96-9C0C-65F82CD3D399

#### Epistagma

Emeljanov, 1999

3799D439-BAA9-5D3C-A442-0A1CE158BF7C

#### * Epistagma (Epistagma) guttulinervis

(Kirschbaum, 1868)

0E3DC5F9-53DC-5220-90B3-B78E9534B928

https://www.gbif.org/species/12210758

https://hoppers.speciesfile.org/otus/25415

##### Distribution

**New data**: Chania: Lefka Ori, on the way to Pachnes peak. Heraklion: Feneromeni; along the River Almyros; Temenos; Archanes; Siva; Petrokefali; between Peri and Platanos; Ammoudara. Lasithi: between Itanos and Vai; Bramiana Dam.

##### Notes

New genus and species for the fauna of Crete. Widespread Palaearctic species (Dimitriev et al. 2022). Collected in pitfall traps left throughout the year, by sweep netting – from June to August up to 1792 m a.s.l.

#### Goniagnathus

Fieber, 1866

7B34AED9-EB70-5D97-A55D-B0274A028932

#### Goniozygotes

Emeljanov, 1999

7B419C79-8642-5AAD-9DB3-5C4AFA0B2EFF

#### Goniagnathus (Goniozygotes) bolivari

(Melichar, 1907)

2219B0DC-5496-5C43-849B-67BCF436D2A3

https://www.gbif.org/species/2042832

https://hoppers.speciesfile.org/otus/25446

##### Distribution

**Literature data**: Chania: Lefka Ori (Lymberakis 2003). **New data**: Chania: Elafonisi; Lefka Ori - Troharis peak; between Therisso and Kaloros; on the way to Pachnes peak; Kouroupitos. Rethymno: Moni Preveli; Exantis; Afentis Christos; Rizikas; Psiloritis; Lohrias; Idaion. Heraklion: Sternes; Koudoumas; Kofinas.

##### Notes

Collected in pitfall traps left throughout the year up to 2347 m a.s.l.

#### Goniagnathus sp.

C1F74B76-EF0C-55EC-AB03-DC67E4C337F9

##### Distribution

**New data**: Herakion: Giouchta Mt.; Gazi; Malia.

##### Notes

Four nymphs collected by sweep net in May and June up to 700 m a.s.l.

#### Koebeliini

Baker, 1897

8A1E91C3-EA42-58E4-893D-676280783BCB

#### Grypotina

Haupt, 1929

B2767FAD-FB91-541A-A7C4-16DDD801505A

#### Grypotellus

Emeljanov, 1999

6A826B1C-A627-53B7-BFF5-29C788D8BD3B

#### * Grypotellus staurus

(Ivanoff, 1885)

D9DECC23-0454-5A94-A178-9833C43DCE2E

https://www.gbif.org/species/2041529

https://hoppers.speciesfile.org/otus/25904

##### Distribution

**New data**: Heraklion: Rufas; Koudoumas.

##### Notes

New species for the fauna of Crete. Widespread Mediterranean species (Dimitriev et al. 2022). Collected in pitfall traps from July to September at 299 – 814 m a.s.l.

#### Limotettigini

Baker, 1915

59C6BFF5-27CC-5F69-AB14-35244C9ED44C

#### Limotettix

Sahlberg, 1871

AFAEBFFC-03BA-574E-AF50-7E8228A19C2E

#### Limotettix

Sahlberg, 1871

07CD14E2-1645-58BC-97E9-27A6F1E95903

#### * Limotettix (Limotettix) striola

(Fallén, 1806)

60ED3943-33BC-5145-8691-EA08BF856974

https://www.gbif.org/species/2043723

https://hoppers.speciesfile.org/otus/651972

##### Distribution

**New data**: Heraklion: on the road between Peri and Platanos.

##### Notes

New species for the fauna of Crete. Widespread Holarctic species (Dimitriev et al. 2022). Collected in single pitfall trap in July at 93 m a.s.l.

#### Macrostelini

Kirkaldy, 1906

0E153250-B130-5D5A-B376-3D3EFF309E36

#### Balclutha

Kirkaldy, 1900

D8A0BA19-4D71-50FB-8B28-8BC26AA07B3B

#### Balclutha cf. frontalis

(Ferrari, 1882)

1E606E17-8163-51B0-9A2E-5BC2B595364D

https://www.gbif.org/species/4482994

https://hoppers.speciesfile.org/otus/26232/overview

##### Distribution

**Literature data**: Chania: no specific location (Koufakis et al. 2024); Platanos (Dlabola 1977). **New data**: Chania: Kedrodasos Beach. Rethymno: Geropotamos bridge. Heraklion: Analipsi; Gazi; between Stalida and Mochlos.

##### Notes

Collected by sweep netting from May to July up to 419 m a.s.l.

#### Balclutha sp.

B92491CB-8EA7-551C-AB1C-3FCA05505DF2

##### Distribution

**New data**: Chania: Stavres, near Zorba Beach. Rethymno: Lavris, after Geropotamos bridge. Heraklion: Gazi; Malia, the path from Stalida to Mochos; Rema Gazanos River.

##### Notes

Collected by sweep netting from May to July from 3 to 419 m a.s.l.

#### Opsiini

Emeljanov, 1962

C9BC7401-9CB1-5AE2-93CC-5BD003E4BA49

#### Circuliferina

Emeljanov, 1962

0511431C-B6C8-5671-8376-BFEF9C4105BD

#### Neoaliturus

Distant, 1918

CFA61062-FE8B-520E-9A3C-DBDD67564CAC

#### Neoaliturus

Distant, 1918

4D93ECE5-D49A-5507-B988-A294314E05EF

#### Neoaliturus cf. fenestratus

(Herrich-Schäffer, 1834)

2725E892-C036-5B36-9EFD-702CCEE1A95D

https://www.gbif.org/species/2048889

https://hoppers.speciesfile.org/otus/27135

##### Distribution

**Literature data**: Chania: Lefka Ori (Lymberakis 2003). **New data**: Chania: Gramvousa Peninsula; Lefka Ori. Heraklion: River Park Almyros; Sternes; Koudoumas; Kofinas.

##### Notes

Collected in pitfall traps left throughout the year up to 2000 m a.s.l. and by sweep netting in May.

#### Circulifer

Zakhvatkin, 1935

C85EAA9D-E6E4-5B53-8BF5-EE92B947AC29

#### Neoaliturus (Circulifer) cf. haematoceps

(Mulsant & Rey, 1855)

161F6812-5D94-5DAB-B1CA-56202002D7B8

https://www.gbif.org/species/2048874

https://hoppers.speciesfile.org/otus/27090

##### Distribution

**Literature data**: no specific location (Dlabola 1977). New data: Chania: Elafonisi; Gavdos Isl. Heraklion: Mires; Koudoumas; Kofinas.

##### Notes

Collected in pitfall traps from March to November up to 639 m a.s.l. and by sweep netting in July.

#### Alituriscus

Emeljanov, 1999

D70C9451-B721-5AAC-B53C-263CDCBE5A00

#### Neoaliturus (Alituriscus) sp.

F616F8E9-AF1F-5BCB-9E92-94DC996A238B

##### Distribution

**New data**: Chania: Askyfou. Rethymno: Exantis, Agios Kyprianos Church; Lochria. Heraklion: Koudoumas; Kofinas; Sternes; Almyros River; Panagia Almyri; Voro

##### Notes

Collected from pitfall traps from May to November at 179-1914 m a.s.l.

#### Opsiina

Emeljanov, 1962

54E229E3-6D0A-5726-A741-F759CCC4C247

#### Opsius

Fieber, 1866

3F2D2E6C-1F29-57D8-BFC6-C38230A25592

#### Opsius stactogalus

Fieber, 1866

C7FDFC3B-F408-51B6-B21C-67D6C565924A

https://www.gbif.org/species/2038171

https://hoppers.speciesfile.org/otus/27462

##### Distribution

**Literature data**: no specific location (de Jong et al. 2014). **New data**: Chania: Lefka Ori; on the road to Pachnes peak. Rethymno: Lavris. Heraklion: In wetland; along the Almyros River; Koudoumas; Komos sand dunes; Karteros Estuary; along the Karteros River; above Mohos.

##### Notes

Collected in pitfall traps from May to December up to 597 m a.s.l. and by sweep netting in June and July up to 1792 m a.s.l.

#### Paralimnini

Distant, 1908

D4D67BD0-34A4-5AD5-B201-453A61F9CE67

#### Paralimnina

Distant, 1908

B2131A3E-E8F2-5C04-8AA5-5596277AFE76

#### Psammotettix

Haupt, 1929

541E28A3-A71B-5DB1-9614-F474AC28AF8D

#### Psammotettix alienus

(Dahlbom, 1850)

8763E27A-7C1F-540B-AEAC-8684F4A61BCD

https://www.gbif.org/species/4483529

https://hoppers.speciesfile.org/otus/28955

##### Distribution

**Literature data**: no specific location (Antonatos et al. 2020). **New data**: Heraklion: Koudoumas.

##### Notes

Collected in pitfall traps from March-April at 178 m a.s.l.

#### Psammotettix sp.

E806644B-0B7A-5566-9861-C6F413B67E2B

##### Distribution

**New data**: Chania: Stavros, near Zorba Beach. Rethymno: Moni Preveli; Psiloritis Mt.. Heraklion: Almyros River; Kofinas; Koudoumas; Omalos Vianou plateau; Roufas, Sternes; Gounes, Karteros; Lavris, Malia, Mires. Lasithi: Xerokampos wetland; Dikti Mt., Katharon.

##### Notes

Collected in pitfall traps and sweep net from May-October up to 1334 m a.s.l.

#### Phlepsiini

Zahniser & Dietrich, 2013

DF08E3B2-A474-5A96-88C7-B32313276DF5

#### Phlepsius

Fieber, 1866

9669B882-10FE-58C3-AB32-B37407021093

#### * Phlepsius ornatus

(Perris, 1857)

2F5FA51F-BA7A-573C-9575-9051F273C1E8

https://www.gbif.org/species/2026452

https://hoppers.speciesfile.org/otus/29963

##### Distribution

**New data**: Heraklion: Koudoumas; Sivas; Komos sand dunes.

##### Notes

New species for the fauna of Crete. The species is widespread in Mediterranean and Central Asia (Dimitriev et al. 2022). Collected in pitfall traps from May to December up to 175 m a.s.l.

#### Phlepsius sp.

63CACC3A-F364-5544-9FFA-FDAEE4572F3D

##### Distribution

**New data**: Heraklion: Koudouma.

##### Notes

Single specimen collected from a pitfall trap in July at 175 m a.s.l.

#### Scaphoideini

Oman, 1943

C4F75C13-D2DF-5304-ACC2-14123C0D63FC

#### Anoplotettix

Ribaut, 1942

ACFBB689-A16B-55D0-89A0-88F58EB24B9D

#### Anoplotettix sp.

BFFB6C32-3659-5288-A776-6DAED5B63004

##### Distribution

**New data**: Rethymno: Petres wetland.

##### Notes

New genus for the fauna of Crete. Single female specimen, collected in a pitfall trap set from May to July.

#### Selenocephalini

Fieber, 1872

D0C4747A-3D06-51AE-8B0A-4ECD9A58F0EB

#### Selenocephalina

Fieber, 1872

CB29CE5E-D83A-5E13-83AF-66781E0E2F4A

#### Selenocephalus

Germar, 1833

7E786129-6502-522C-8596-067F2397F486

#### ** Selenocephalus cf. conspersus

(Herrich-Schäffer, 1834)

19F5E5ED-4C5C-5758-88A7-D21A4E9944CC

https://www.gbif.org/species/2030181

https://hoppers.speciesfile.org/otus/31449/overview

##### Distribution

**New data**: Chania: Gramvousa Peninsula. Heraklion: Koudoumas

##### Notes

New species for Greece. European species (Dimitriev et al. 2022). Collected in pitfall traps from April to July from 419 to 812 m a.s.l.

#### ** Selenocephalus cf. deserticola

Linnavuori, 1962

841AAECA-D3E5-55A7-9112-6045C7313B4A

https://www.gbif.org/species/2030208

https://hoppers.speciesfile.org/otus/31455/overview

##### Distribution

**New data**: Lasithi: Xerokampos.

##### Notes

New species for Greece. The species is described from Israel and, apart from its type locality, has only been found in Egypt (Dlabola 1974b). Single specimen collected in pitfall trap from the end of May till the begining of August at 2 m a.s.l.

#### * Selenocephalus obsoletus

(Germar, 1817)

C4308F72-9051-5EB1-AD68-DFB01AF4C018

https://www.gbif.org/species/4483172

https://hoppers.speciesfile.org/otus/31475

##### Distribution

**New data**: Chania: Gavdos isl. Rethymno: Exantis. Heraklion: Koudoumas.

##### Notes

New species for the fauna of Crete. The species is widespread in the Western Palaearctic (Dimitriev et al. 2022). Collected in pitfall traps from April to August from 8 to 523 m a.s.l.

#### * Selenocephalus pallidus

Kirschbaum, 1868

7B00E9E1-3097-5D1A-98C4-ECE9A2B27CD6

https://www.gbif.org/species/2030187

https://hoppers.speciesfile.org/otus/31485

##### Distribution

**New data**: Chania: Gavdos Isl. Rethymno: Kouroutes. Heraklion: Koudoumas; Kofinas. Lasithi: Mochlos.

##### Notes

New species for the fauna of Crete. The species is distributed in southern Mediterranean (Dimitriev et al. 2022). Collected in pitfall traps from March to September up to 729 m a.s.l. and by sweep netting in July.

#### * Selenocephalus stenopterus

Signoret, 1880

A915D336-F193-566A-BB53-D11074C2A1C4

https://www.gbif.org/species/2030197

https://hoppers.speciesfile.org/otus/31488/overview

##### Distribution

**New data**: Heraklion: Koudoumas, Kofinas.

##### Notes

New species for the fauna of Crete. The species is known from Italy, north-easten Mediterranean, Crimea and Central European Russia (Dimitriev et al. 2022). Caught in pitfall traps from June to August from 523-812 m a.s.l.

#### Selenocephalus sp.

D789FB9B-B40E-568B-B7F8-938505145C22

##### Distribution

**New data**: Chania: Kouroupitos; Gavdos Isl.; Lefka Ori, Trocharis peak; Gramvousa Peninsula. Rethymno: Rizikas. Heraklion: Almyros River; Livadopa; Giouchtas Mt.; Koudoumas. Lasithi: Ierapetras, Archaies dexamenes ierapetras; Istro.

##### Notes

Collected in pitfall traps from January to October at 2 - 1900 m a.s.l.

#### Deltocephalinae undet.

AE13A3C9-5AB3-5594-BC73-823D890F724A

##### Notes

There were 12 males, 72 females (six of which were Athysanini) and 12 nymphs (six of which were Fieberiellini and four of which were Goniagnathini) from various locations on the Island.

#### Dorycephalinae

Oman, 1943

CCC031CD-2859-5137-8CFC-0D9D61D50642

#### Eupelicini

Sahlberg, 1871

42244D5C-F8A8-5FB3-B397-061825C2AEFD

#### Eupelicina

Sahlberg, 1871

FEBBAC8D-A518-576F-8B28-FD8B4B35CB06

#### Eupelix

Germar, 1821

2F7A6786-240D-51EF-B91C-4DFB28A2A277

#### * Eupelix cuspidata

(Fabricius, 1775)

45B349E4-9FE0-5C0E-9B40-246664D01F99

https://www.gbif.org/species/2028442

https://hoppers.speciesfile.org/otus/25152

##### Distribution

**New data**: Chania: Gavdos Isl.; Elafonisi. Rethymno: Exantis. Heraklion: Koudoumas.

##### Notes

New record for the fauna of Crete. Widespread Palaearctic species (Dimitriev et al. 2022). Collected in soil traps left throughout the year up to 1334 m a.s.l.

#### Eurymelinae

Amyot & Audinet-Serville, 1843

07FF74B9-2A5A-5899-9E65-2456024F0C05

#### Idiocerini

Baker, 1915

A4001EE1-3286-52BB-993C-801BFAF32B67

#### Idiocerina

Baker, 1915

C119F72C-76CF-511C-9CB9-4D613AFBF862

#### Acericerus

Dlabola, 1974

335D3645-CB9D-51AA-B73C-8DFCC6C32D9B

#### * Acericerus vittifrons

(Kirschbaum, 1868)

98D9C8D6-4726-5956-97E7-E07847CC8653

https://www.gbif.org/species/4484047

https://hoppers.speciesfile.org/otus/32374

##### Distribution

**New data**: Chania: Troharis peak, Lefka Ori. Rethymno: Psiloritis above Anogia on the E4 path; Heraklion: Omalos plateau.

##### Notes

New genus and species for the fauna of Crete. The species is widespread in Europe (Dimitriev et al. 2022). Collected in pitfall traps set from January to March and a single collection from September to 2347 m a.s.l. and by sweep netting in the second half of August on *Acer sempervirens* at about 1100 m above sea level.

#### Acericerus sp.

26FD1EAD-ADF8-54A8-9B6E-5CD98F594D2F

##### Distribution

**New data**: Rethymno: Psiloritis Mt.

##### Notes

Two nymphs collected by sweep netting in August.

#### Bugraia

Koçak, 1981

C63ACA30-8EF0-5CF0-ADEA-1592CF78E863

#### Bugraia sp.

51AB54EB-2F4D-570B-8F27-21CF3B7C16D9

##### Distribution

**New data**: Chania: Gavdos, Gavdopoula Isl.; Gavdos Isl., Kedres wetland; Lefka Ori Mt. Trocharis peak; Falasarna wetland; Stavros, near Zorba Beach.

##### Notes

New genus for the fauna of Crete. Seven females collected in pitfall traps from March to June and by sweep net from June to September up to 1900 m a.s.l.

#### Macropsini

Evans, 1936

90C7CD4E-59AD-56E6-9522-3FEA1BB73571

#### Macropsis

Lewis, 1834

C30221F0-4905-5898-AE63-03656AA67B4D

#### Macropsis

Lewis, 1834

9B1A2F6E-9CC3-5C3F-97B1-956C663A5BE2

#### ** Macropsis (Macropsis) brabantica

Wagner, 1964

A4BE636F-913B-501F-9CCF-DB2BCD4FB063

https://www.gbif.org/species/2039839

https://hoppers.speciesfile.org/otus/33545

##### Distribution

**New data**: Heraklion: Kofinas, Sternes.

##### Notes

New species for the fauna of Greece. A rare species with unclear distribution. So far, it has been found in the Netherlands, Germany and European Russia (Dimitriev et al. 2022). Collected in pitfall traps from July to September up to 786 m a.s.l.

#### ** Macropsis (Macropsis) fuscula

(Zetterstedt, 1828)

92129B5F-9C6A-5F69-810F-B8F07791DCFE

https://www.gbif.org/species/4483855

https://hoppers.speciesfile.org/otus/33599

##### Distribution

**New data**: Heraklion: Kofinas.

##### Notes

New species for the fauna of Greece. Palaearctic species registered as non-indigenous in North America (Hamilton 1983, Dimitriev et al. 2022). Collected from pitfall traps in July at 638 m a.s.l.

#### Macropsis (Macropsis) heracleionica

Dlabola, 1967

E4C9CE7E-3752-5736-8A1F-E390A83E85E0

https://www.gbif.org/species/2039857

https://hoppers.speciesfile.org/otus/33626

##### Distribution

**Literature data**: no specific locality (Nast 1972) Rethymno: Amari; Psiloritis (Dlabola 1967)**. New data**: Heraklion: Kofinas.

##### Notes

Considered endemic to Crete. Collected in pitfall traps from July to September up to 786 m a.s.l.

#### Megophtalminae

Kirkaldy, 1906

6528ABED-764C-5E17-A1EB-41B9F8335CE8

#### Agalliini

Kirkaldy, 1901

621D1B35-8882-5863-B472-6BA36EE7433A

#### Agalliina

Kirkaldy, 1901

0F2DB610-8C0C-5267-AA0B-91F9384449C6

#### Agallia

Curtis, 1833

B4E534EB-EE55-5B41-A05A-F2B06B492723

#### ** Agallia brachyptera

(Boheman, 1847)

9677622B-D6CE-5EE7-A1E0-C700A8D627C9

https://www.gbif.org/species/4482818

https://hoppers.speciesfile.org/otus/38776/overview

##### Distribution

**New data**: Chania: Kournas Lake.

##### Notes

New species for Greece and new genus for Crete. Widespread Western Palaearctic species (Dimitriev et al. 2022). Single specimen from a pitfall trap from the beginning of May and end of June at 24 m a.s.l.

#### Agallia sp.

5C260E1B-8110-58D9-B78D-683D0F556FAC

##### Distribution

**New data**: Lasithi: Xerokampos.

##### Notes

Collected in pitfall traps from late May to late November at 1-2 m a.s.l.

#### Anaceratagallia

Zakhvatkin, 1946

181B5D5D-877C-5052-8A74-FB8CE9C7C107

#### Anaceratagallia

Zakhvatkin, 1946

8BA23976-26AA-5200-B6FA-45E708FF3854

#### * Anaceratagallia (Anaceratagallia) cf. glabra

Dmitriev, 2020

490018CA-D7FF-5524-BCD1-E41D0993E963

https://www.gbif.org/species/10732013

https://hoppers.speciesfile.org/otus/615713

##### Distribution

**New data**: Chania: Gramvousa Peninsula; Lefka Ori - bellow Pachnes peak; Gavdos Isl. Rethymno: Moni Preveli; Agios Titos; Kardak; Kouroutes; Amari. Heraklion: along the Almyros River; between Peri and Platanos; Siva; Rufas; Kofinas; Sternes; Koudoumas; Amoudara. Lasithi: Xirokampos; Krustas; Paheia Amos.

##### Notes

New species for the fauna of Crete. Widespread Western Palaearctic species (Dimitriev et al. 2022). It was found in pitfall traps for the periods: January – February, March – May, June – August and November – May at altitudes from 0 to 786 m above sea level.

#### Anaceratagallia (Anaceratagallia) sp.

69EE8D64-FEB4-58B4-A18A-1B20D78643D6

##### Distribution

**New data**: Heraklion: Monofatsiou Kofinas.

##### Notes

Single male specimen collected in a pitfall trap from January to March at 717 m a.s.l.

#### Megophthalmini

Kirkaldy, 1906 [1859]

1FCCA459-3139-56A5-94D9-1331E4E5C6E2

#### Megophthalmus

Curtis, 1833

290BAC22-499B-5C9E-B227-360771A93174

#### * Megophthalmus scabripennis

Edwards, 1915

DEDC2BBD-EDF6-5188-AEE3-4ED2C13CE526

https://www.gbif.org/species/2033770

https://hoppers.speciesfile.org/otus/39546

##### Distribution

**New data**: Chania: Gavdos Isl; Gavdopula Isl.; Gramvousa Peninsula. Rethymno: Moni Preveli; Agios Titos. Heraklion: Giouchtas Mt.; Kofinas; Sternes; Keratokampos. Lassithi: Topleni Monastery.

##### Notes

A new species for the fauna of Crete. Widespread Western Palaearctic species (Dimitriev et al. 2022). Collected in pitfall traps from March to December up to 721 m a.s.l. and by sweep netting in May at 800 m a.s.l.

#### Typhlocybinae

Kirschbaum, 1868

FD896A2A-7CDB-5988-A270-5A385FDF5A6F

#### Erythroneurini

Young, 1952

9E0A3B10-1CFE-5771-BACE-34406BF1C514

#### Zyginidia

Haupt, 1929

2F5EA31A-709C-5C76-A552-8635DAADAF48

#### Zyginidia pullula

(Boheman, 1845)

9EF32038-3EA9-5106-AC3C-AEF294D6FC5F

https://www.gbif.org/species/2027339

https://hoppers.speciesfile.org/otus/46566

##### Distribution

**Literature data**: Chania: Chania (Tsagkarakis et al. 2018). **New data**: Heraklion: Mires.

##### Notes

Collected by sweep netting in late July at 77 m a.s.l. on herbaceous vegetation under olive trees in an olive grove.

#### Typhlocybini

Kirschbaum, 1868

02E0A90A-EC83-5938-86A5-7AA0B8DE7832

#### Eupteryx

Curtis, 1831

7C320E91-0144-5A1B-A4E5-8090E156490C

#### Eupteryx

Curtis, 1831

D030F4DA-4A5D-536F-BC66-49C400D9C979

#### Eupteryx (Eupteryx) melissae

Curtis, 1837

51CE413A-05C4-58D2-8426-27E7B4655201

https://www.gbif.org/species/5985079

https://hoppers.speciesfile.org/otus/47330

##### Distribution

**Literature data**: Chania: Chania (Tsagkarakis et al. 2018). Heraklion: Knossos (Dworakowska 1972). Lasithi: Myti (Dlabola 1977). **New data**: Chania: Agia Irini gorge, on E4 path.

##### Notes

Collected by sweep netting in herbaceous vegetation in early June in the upper part of the Agia Irini gorge at 553 m a.s.l.

#### Eupteryx (Eupteryx) zelleri

(Kirschbaum, 1868)

CC918F2E-74C5-5175-872A-DA108421B925

https://www.gbif.org/species/5984890

https://hoppers.speciesfile.org/otus/47444

##### Distribution

**Literature data**: Chania: no specific location. Heraklion: Knossos (Dworakowska 1972). **New data**: Heraklion: along the Almyros River; Almyros River Park.

##### Notes

Collected from May to July by sweep netting on *Origanum* sp. up to 327 m a.s.l.

#### Eupteryx sp.

987D7D38-500E-5201-9761-F3038F7964FB

##### Distribution

**New data**: Heraklion: Almyros River.

##### Notes

Two male and two female specimens, collected in pitfall traps from May to October.

#### Eurhadina

Haupt, 1929

24BC9CC7-F1FD-570C-B08F-7BF6CCC9B456

#### Eurhadina

Haupt, 1929

96B5E1B2-85F2-5648-A24E-E17D3E2F22DA

#### ** Eurhadina (Eurhadina) saageri

Wagner, 1937

4C2FF1BB-944E-5EF5-BF3B-2C685E29DBC0

https://www.gbif.org/species/2049147

https://hoppers.speciesfile.org/otus/47524

##### Distribution

**New data**: Rethymno: Psiloritis, above Anogia on E4 path.

##### Notes

New species for Greece and new genus for Crete. Collected by sweep netting in late August at 1100 m a.s.l. on trees of the genus *Quercus*.

## Discussion

From the deposited collection in the Natural History Museum of Crete, 93 species of the suborder Auchenorrhyncha (Insecta, Hemiptera) from the Island of Crete were prepared and identified, belonging to 11 families. They represent 52.5% of the species known for Crete. This shows that, despite the unsuitable main collection method for the group (soil traps) and the short collection period using the most effective method (mowing with an entomological bag), the richness of the collection is relatively high. In addition, pitfall traps are not a suitable method for the group, but nevertheless, we were surprised by the diversity of species that have accidentally fallen into the traps - a good quantity of epigeobionts like the species of genus *Anoscopus* and *Aphrodes*. Of the identified species, 37 are new to the Island of Crete and 10 are new to Greece and 13 new genera to Crete. Approximately 14% of the identified species are endemic, which is indicative of the high percentage of endemism on the Island and its great conservation value. Nine of these species are endemic to Crete, while four are endemic to Greece. It is important to note that the collection is devoid of non-indigenous species, a fact that gives rise to a number of questions that require further research.

According to the available data, the distribution of species by family is not uniform in the two infraorders of Auchenorrhyncha. In the Cicadomorpha infraorder, the Cicadellidae family dominates in terms of species number – 101, as expected given that it is the most species-rich family in the group. The Cicadidae family is represented by three species, the Aphrophoridae family by five and the Cercopidae family by only three, making a total of 112 species in Cicadomorpha. In the infraorder Fulgoromorpha, the most species-rich family is Delphacidae, as expected given that it is also the largest family in this group. In Crete, the Delphacidae family numbers 32 species, followed by the Issidae family with 14 species. Third place goes to the Cixiidae family with 11 species. The Achilidae family is represented by four species, the Tettigometridae family by two and the Meenoplidae and Ricaniidae families by one each, making a total of 65 species in Fulgoromorpha. In terms of distribution, the situation is similar for most families to that in Europe as a whole. However, the Achilidae and Issidae families undoubtedly have a greater richness. However, some families that are common in European fauna are absent, including Membracidae, Ulopidae, Caliscelidae, Dictyopharidae and Flatidae. Published information on species on the Island of Crete was compared with information for Europe as a whole. This revealed an unusual ratio between the two infraorders, Cicadomorpha and Fulgoromorpha, within the group. Due to the greater species richness of Cicadomorpha on a global and European scale, there should be more Cicadomorpha than Fulgoromorpha. This is exactly what is observed in European species: 65% (1,039 species) are Cicadomorpha and 35% (550 species) are Fulgoromorpha. Prior to this study, the species in Crete exhibited a somewhat atypical ratio of 60% Cicadomorpha (87 species) to 40% Fulgoromorpha (58 species). This is mainly due to the thorough studies of the Delphacidae family by Asche, Remane, Hoch and Drosopoulos in the early 1980s. This is also the family with the largest number of known Fulgoromorpha species for Crete. The results of the present study mean that this ratio is beginning to approach the European one. Thirty-seven new species have been added to the fauna of Crete, 30 of which are from the infraorder Cicadomorpha and seven from Fulgoromorpha.

## Supplementary Material

DA69DA9C-C3FC-548E-8BC4-080312795BCB 10.3897/BDJ.14.e177170.suppl1 Supplementary material 1 List of Cretan species of Auchenorrhyncha Data type check-list Brief description A checklist of Cretan species of Auchenorrhyncha, based on literature data and specimens from the Heraklion Museum. File: oo_1455384.pdf https://binary.pensoft.net/file/1455384 Angelova, R., Gjonov, I., Trichas, A.
