# Supplementary material for: Auchenorrhyncha (Hemiptera) from Crete deposited at the Natural History Museum of Crete
Source: Biodivers Data J. 2026 Jan 30;14:e177170. doi: 10.3897/BDJ.14.e177170 (PMC12881913; doi:10.3897/BDJ.14.e177170)
Supplement: Supplementary material 1 — List of Cretan species of Auchenorrhyncha [file bdj-14-e177170-s001.pdf]

List of Cretan species of Auchenorrhyncha

		in NHMC collection
**Infraorder Fulgoromorpha Evans, 1946**		
**Superfamily Fulgoroidea Latreille, 1807**		
**Family Achilidae Stål, 1866**		
1	_Cixidia malickii_ Asche, 2015	
2	_Cixidia skaloula_ Asche, 2015	*
3	_Cixidia advena_ (Spinola, 1839)	
4	_Neomenocria creticola_ Asche, 2015	*
**Family Issidae Spinola, 1839**		
5	_Acrestia suturalis_ (Fieber, 1877)	*
6	_Agalmatium bilobum_ (Fieber, 1877)	*
7	_Agalmatium flavescens_ (Olivier, 1791)	
8	_Clybeccus declivus_ Dlabola, 1986	*
9	_Falcidius aptera_ (Fabricius, 1794)	
10	_Latilica oertzeni_ (Matsumura, 1910)	*
11	_Latilica tunetana_ (Matsumura, 1910)	*
12	*_Mycterodus colossicus_ (Dlabola, 1987)	*
13	_Mycterodus idomeneus_ Dlabola 1984	*
14	_Mycterodus lapaceki_ Dlabola 1984	*
15	_Mycterodus pallens_ Stål, 1861	*
16	_Mycterodus wittmeri_ Dlabola, 1974	*
17	_Rhissolepus insulanus_ (Dlabola, 1982)	*
18	**_Tshurtshurnella pythia_ Dlabola, 1979	*
**Family Ricaniidae Amyot & Audinet-Serville, 1843**		
19	_Ricania hedenborgi_ Stål, 1865	
**Family Tettigometridae Germar, 1821**		
20	*_Tettigometra impressifrons_ Mulsant & Rey, 1855	*
21	**_Tettigometra picta_ Fieber, 1865	*
**Superfamily Meenoploidea Fieber, 1872**		
**Family Meenoplidae Fieber, 1872**		
22	_Nisia atrovenosa_ (Lethierry, 1888)	
**Superfamily Delphacoidea Leach, 1815**		
**Family Cixiidae Spinola, 1839**		
23	_Cixius carniolicus_ Wagner, 1939	
24	_Cixius pallipes_ Fieber, 1876	
25	*_Cixius wagneri_ China, 1942	*
26	_Hyalesthes obsoleta_ Signoret, 1865	*
27	_Pentastira demaculata_ Dlabola, 1989	*
28	_Pentastira major_ Kirschbaum, 1868	
29	_Pentastiridius leporinus_ (Linnaeus, 1761)	*
30	**_Pentastiridius_ cf. _ovatus_ (Metcalf, 1955)	*
31	_Reptalus quinquecostatus_ (Dufour, 1833)	*
32	_Reptalus panzeri_ (Löw, 1883)	*
33	_Tachycixius creticus_ Dlabola, 1974	*
**Family Delphacidae Leach, 1815**		
**Subfamily Asiracinae Motschulsky, 1863**		
34	_Asiraca clavicornis_ (Fabricius, 1794)	*
**Subfamily Delphacinae Leach, 1815**		
35	_Bostaera bolivari_ (Melichar, 1909)	
36	_Chloriona ponticana_ Asche, 1982	
37	_Chloriona unicolor_ (Herrich-Schäffer, 1835)	*
38	_Conomelus odryssius_ Dlabola, 1965	
39	_Delphacodes mulsanti_ (Fieber, 1866)	
40	_Delphax inermis_ (Ribaut, 1934)	
41	_Distantinus putoni_ (Costa, 1888)	

List of Cretan species of Auchenorrhyncha

		in NHMC collection
42	*Ditropis pteridis* (Spinola, 1839)	
43	*Euconomelus lepidus* (Boheman, 1847)	
44	*Euidopsis truncata* Ribaut, 1948	
45	*Eurysa duffelsi* Drosopoulos & Asche, 1984	*
46	**Eurysa lineata* (Perris, 1857)	*
47	*Flastena fumipennis* (Fieber, 1866)	
48	*Iubsoda stigmatica* (Melichar, 1897)	
49	*Javasella dubia* (Kirschbaum, 1868)	
50	*Javasella obscurella* (Boheman, 1847)	
51	*Laodelphax striatellus* (Fallén, 1826)	
52	*Leptofloria leptosoma* (Flor, 1861)	
53	*Matutinella putoni* (Costa, 1888)	
54	*Megadelphax sordidula* (Stål, 1853)	
55	*Megamelodes quadrimaculatus* (Signoret, 1865)	
56	**Muirodelphax aubei* (Perris, 1857)	*
57	*Pseudaraeopus lethierryi* (Mulsant & Rey, 1879)	*
58	*Sogatella vibix* Haupt, 1927	
59	*Toya tuberculosa* (Distant, 1916)	
60	*Toya propinqua* (Fieber, 1866)	*
61	*Tropidocephala tuberipennis* Mulsant & Ray, 1855	*
**Subfamily Kelisiinae Wagner, 1963**		
62	*Kelisia brucki* Fieber, 1878	
63	*Kelisia creticola* Asche, 1982	*
64	*Kelisia guttula* (Germar, 1818)	
65	*Kelisia ribauti* Wagner, 1938	
**Infraorder Cicadomorpha Evans, 1946**		
**Superfamily Cicadoidea Latreille, 1802**		
**Family Cicadidae Latreille, 1802**		
66	*Cicada cretensis* Quartau & Simões , 2005	*
67	*Oligoglena carayoni* Boulard 1982	*
68	*Pagiphora aschei* Kartal 1978	*
**Superfamily Cercopoidea Leach, 1815**		
**Family Aphrophoridae Amyot & Audinet-Serville, 1843**		
69	*Aphrophora alni* (Fallén, 1805)	
70	**Lepyronia coleoptrata* (Linnaeus, 1758)	*
71	*Neophilaenus campestris* (Fallén, 1805)	*
72	*Philaenus signatus* (Melichar, 1896)	*
73	*Philaenus spumarius* (Linnaeus, 1758)	*
**Family Cercopidae Leach, 1815**		
74	**Cercopis sanguinolenta* (Scopoli, 1763)	*
75	**Haematoloma dorsatum* (Ahrens, 1812)	*
76	*Triecphorella geniculata* (Horvath, 1831)	
**Superfamily Membracoidea Rafinesque, 1815**		
**Family Cicadellidae Latreille, 1825**		
**Subfamily Aphrodinae Haupt, 1927 [1859]**		
77	*Anoscopus albifrons* (Linnaeus, 1758)	*
78	*Anoscopus albiger* (Germar, 1821)	
79	**Anoscopus flavostriatus* (Donovan, 1799)	*
80	***Anoscopus gorloppus* Guglielmino & Bückle, 2015	*
81	***Anoscopus samuricus* (Tshmir, 1977)	*
82	*Aphrodes bicincta* (Schrank, 1776)	*
83	*Aphrodes carinatus* (Stål, 1864)	
84	***Aphrodes diminuta* Ribaut, 1952	*
85	*Aphrodes makarovi* Zachvatkin, 1948	*

List of Cretan species of Auchenorrhyncha

		in NHMC collection
	**Subfamily Deltocephalinae Fieber, 1869**	
86	***Aconura jakowlefi* Lethierry, 1876	*
87	*Aconurella prolixa* (Lethierry, 1885)	
88	*Allygidius atomarius* (Fabricius, 1794)	
89	**Allygus modestus* Scott, 1876	*
90	*Balclutha frontalis* (Ferrari, 1882)	*
91	*Balclutha punctata* (Fabricius, 1775)	
92	*Balclutha rhenana* Wagner, 1939	
93	**Chiasmus conspurcatus* (Perris, 1857)	*
94	*Cicadula placida* (Horváth, 1897)	
95	*Cicadula quadrinotata* (De Villers, 1789)	
96	*Cicadulina bipunctata* (Melichar, 1904)	
97	*Concavifer bolkarensis* Kartal, 1982	
98	*Conosanus obsoletus* (Kirschbaum, 1858)	*
99	*Eohardya fraudulenta* (Horváth, 1903)	*
100	**Epistagma guttulinervis* (Kirschbaum, 1868)	*
101	**Eupelix cuspidata* (Fabricius, 1775)	*
102	*Euscelidius mundus* (Haupt, 1927)	
103	*Euscelidius variegatus* (Kirschbaum, 1858)	*
104	**Euscelis incisa* (Kirschbaum, 1858)	*
105	*Euscelis lineolata* Brullé, 1832	*
106	*Euscelis ohausi* Wagner, 1939	*
107	*Exitianus capicola* (Stål, 1855)	*
108	*Fieberiella ida* Dlabola, 1965	
109	*Fieberiella kritiella* Dlabola 1989	*
110	*Fieberiella malickana* Dlabola, 1994	
111	*Grypotes puncticollis* (Herrich-Schäffer, 1834)	
112	**Grypotellus staurus* (Ivanoff, 1885)	*
113	*Goniagnathus bolivari* (Melichar, 1907)	*
114	**Limotettix striola* Fallén, 1806	*
115	*Macrosteles laevis* (Ribaut, 1927)	
116	*Macrosteles ossiannilssoni* Lindberg, 1963	
117	*Maiestas schmidtgeni* (W.Wagner, 1939)	*
118	*Mavromoustaca macchiae* (Lindberg, 1948)	
119	*Melillaia desbrochersi* (Lethierry, 1889)	*
120	*Neoaliturus dubiosus* (Matsumura, 1908)	
121	*Neoaliturus fenestratus* (Herrich-Schäffer, 1834)	*
122	*Neoaliturus haematoceps* (Mulsant & Rey, 1855)	*
123	*Nesoclutha erythrocephala* (Ferrari, 1882)	
124	*Opsius stactogalus* Fieber, 1866	*
125	*Phlepsius intricatus* (Herrich- Schaffer)	
126	**Phlepsius ornatus* Perris, 1857	*
127	*Phlogotettix cyclops* (Mulsant & Rey, 1855)	
128	*Platymetopius guttatus* Fieber, 1869	
129	**Proceps acicularis* Mulsant & Rey, 1855	*
130	*Psammotettix alienus* (Dahlbom 1850)	*
131	*Psammotettix notatus* (Melichar, 1896)	
132	***Selenocephalus* cf. *conspersus* (Herrich-Schäffer, 1834)	*
133	***Selenocephalus deserticola* Linnavuori, 1962	*
134	*Selenocephalus obsoletus* (Germar, 1817)	*
135	**Selenocephalus pallidus* (Kirschbaum, 1868)	*
136	**Selenocephalus stenopterus* Signoret, 1880	*
137	*Stymphalus rubrolineatus* (Stål, 1855)	
138	*Synophropsis lauri* (Horváth, 1897)	*

List of Cretan species of Auchenorrhyncha

	in NHMC collection
139 *Thamnotettix creticus* Dlabola, 1974	
140 *Thamnotettix dilutior* (Kirschbaum, 1868)	
141 *Thamnotettix minoidis* Dlabola, 1974	*
142 **Thamnotettix zelleri* Kirschbaum, 1868	*
**Subfamily Eurymelinae Amyot & Audinet-Serville, 1843**	
143 **Acericerus vittifrons* (Kirschbaum, 1868)	*
144 *Idiocerus vicinus* Melichar, 1898	
145 ***Macropsis brabantica* Wagner, 1964	*
146 ***Macropsis fuscula* (Zetterstedt, 1828)	*
147 *Macropsis heracleionica* Dlabola, 1967	*
148 *Tremulicerus mesopyrrhus* (Kirschbaum, 1868)	
**Subfamily Megophthalminae Kirkaldy, 1906**	
149 ***Agallia brachyptera* (Boheman, 1847)	*
150 **Anaceratagallia* cf. *glabra* Dmitriev, 2020	*
151 *Anaceratagallia venosa* (Fourcroy, 1785)	
152 *Austroagallia sinuata* (Mulsant & Rey, 1835)	
153 **Megophtalmus scabripennis* Edwards, 1915	*
**Subfamily Typhlocybinae Kirschbaum, 1868**	
154 *Edwardsiana cretica* Dworakowska, 1971	
155 *Edwardsiana iranicola* Zachvatkin, 1947	
156 *Edwardsiana tersa* (Edwards, 1914)	
157 *Eupteryx cypria* (Ribaut, 1948)	
158 *Eupteryx gyaurdagicus* Dlabola, 1957	
159 *Eupteryx insulana* (Ribaut, 1948)	
160 *Eupteryx melissae* Curtis, 1837	*
161 *Eupteryx urticae* (Fabricus, 1926)	
162 *Eupteryx zelleri* (Kirschbaum, 1868)	*
163 ***Eurhadina saageri* Wagner, 1937	*
164 *Fagocyba cruenta* (Herrich-Schäffer, 1838)	
165 *Fruticidia bisignata* (Mulsant & Rey. 1855)	
166 *Hauptidia provincialis* (Ribaut, 1931)	
167 *Hauptidia soosi* (Dlabola, 1952)	
168 *Hebata affinis* (Nast, 1937)	
169 *Hebata solani* (Curtis, 1846)	
170 *Lindbergina cretica* Asche 1980	
171 *Ribautiana tenerrima* (Herrich-Schäffer, 1834)	
172 *Tamaricella ribauti* (Zachvatkin, 1947)	
173 *Tamaricella tamaricis* (Puton, 1872)	
174 *Zygina angusta* Lethierry, 1874	
175 *Zygina roseipennis* (Tollin, 1851)	
176 *Zyginidia scutellaris* (Herrich-Schäffer, 1838)	
177 *Zyginidia pullula* (Boheman, 1845)	*

Excluded from the list

Tettigometra pallipes (Lucas, 1853)
Aestuansella aestuans (Fabricius, 1794)
Cicada orni Linnaeus, 1758
Dimissalna dimissa (Hagen, 1856)
Oligoglena tibialis (Panzer, 1798)

* New record for Crete
** New record for Greece
